# The Role of Iron in the P-Acquisition Mechanisms of the Unicellular N_2_-Fixing Cyanobacteria *Halothece* sp., Found in Association With the Mediterranean Seagrass *Posidonia oceanica*

**DOI:** 10.3389/fmicb.2019.01903

**Published:** 2019-08-22

**Authors:** Víctor Fernández-Juárez, Antoni Bennasar-Figueras, Antonio Tovar-Sanchez, Nona Sheila R. Agawin

**Affiliations:** ^1^Marine Ecology and Systematics (MarEs), Department of Biology, Universitat de les Illes Balears (UIB), Palma, Spain; ^2^Grup de Recerca en Microbiologia, Departament de Biologia, Universitat de les Illes Balears (UIB), Palma, Spain; ^3^Department of Ecology and Coastal Management, Andalusian Institute for Marine Sciences, ICMAN (CSIC), Cádiz, Spain

**Keywords:** *Halothece* sp. PCC 7418, *Posidonia oceanica*, alkaline phosphatase, N_2_ fixation, PO_4_^3-^-Fe uptake, iron, PhoD, PhoU

## Abstract

*Posidonia oceanica*, an endemic seagrass of the Mediterranean Sea harbors a high diversity of N_2_-fixing prokaryotes. One of these is *Halothece* sp., a unicellular N_2_-fixing cyanobacteria detected through *nifH* analysis from the epiphytes of *P*. *oceanica*. The most related strain in culture is *Halothece* sp. PCC 7418 and this was used as the test organism in this study. In the Mediterranean Sea, phosphorus (P) and iron (Fe) can be the major limiting nutrients for N_2_ fixation. However, information about the mechanisms of P-acquisition and the role of metals (i.e., Fe) in these processes for N_2_-fixing bacteria is scarce. From our genomic analyses of the test organism and other phylogenetically related N_2_-fixing strains, *Halothece* sp. PCC 7418 is one of the strains with the greatest number of gene copies (eight copies) of alkaline phosphatases (APases). Our structural analysis of PhoD (alkaline phosphatase type D) and PhoU (phosphate acquisition regulator) of *Halothece* sp. PCC 7418 showed the connection among metals (Ca^2+^ and Fe^3+^), and the P-acquisition mechanisms. Here, we measured the rates of alkaline phosphatase activity (APA) through MUF-P hydrolysis under different combinations of concentrations of inorganic P (${PO}_{4}^{3-}$) and Fe in experiments under N_2_-fixing (low ${\mathrm{NO}}_{3}^{-}$ availability) and non-N_2_ fixing (high ${\mathrm{NO}}_{3}^{-}$ availability) conditions. Our results showed that APA rates were enhanced by the increase in Fe availability under low levels of ${PO}_{4}^{3-}$, especially under N_2_-fixing conditions. Moreover, the increased ${PO}_{4}^{3-}$-uptake was reflected in the increased of the P-cellular content of the cells under N_2_ fixation conditions. We also found a positive significant relationship between cellular P and cellular Fe content of the cells (*r*^2^ = 0.71, *p* < 0.05). Our results also indicated that Fe-uptake in *Halothece* sp. PCC 7418 was P and Fe-dependent. This study gives first insights of P-acquisition mechanisms in the N_2_-fixing cyanobacteria (*Halothece* sp.) found in *P*. *oceanica* and highlights the role of Fe in these processes.

## Introduction

*Posidonia oceanica* is an endemic seagrass in the Mediterranean Sea, forming extensive meadows with valuable established key ecological services: high primary productivity, as a carbon sink, as a habitat and nursery for a variety of micro- and macro-organisms, as sediment stabilizers, as buffers for ocean acidification, and as an important site for biogeochemical processes (e.g., nitrogen cycles) (Gutiérrez et al., 2012; Campagne et al., 2015; Agawin et al., 2016). Atmospheric nitrogen (N_2_) fixation associated with *P*. *oceanica* meadows are similar in rates or even higher than tropical seagrasses and may play a key role in maintaining the high productivity of the *P*. *oceanica* in oligotrophic waters (Agawin et al., 2016, 2017). N_2_ fixation in *P*. *oceanica* is carried out by microorganisms called diazotrophs that can be found on the surface of the leaves, roots, and rhizomes (epiphytic population) or even on the inside of the roots (endophytic population) (Sohm et al., 2011; Agawin et al., 2019). Among the diazotrophic prokaryotes, a huge variety of diazotrophic cyanobacteria have been detected based on the sequence analysis of *nifH* gene (gene coding for the nitrogenase enzyme responsible for the N_2_ fixation) on the leaves of *P*. *oceanica* (Agawin et al., 2016, 2017).

In general, cyanobacteria are key components in the marine food web, contributing significantly to primary production in oligotrophic oceans (Agawin et al., 2000; Herrero and Flores, 2008). Compared with other phytoplankton taxa, cyanobacteria have elevated ratio of nitrogen (N):phosphorus (P) (a molar ratio above 25 compared with the general Redfield ratio of 16 in marine phytoplankton) and can be a consequence of having two light-harvesting complexes (Redfield, 1934; Geider and La Roche, 2002; Quigg et al., 2011). Changes affecting the N:P ratios in their environment by limiting concentration of N or P, could change their N:P tissue composition and may have consequences in their adaptation and survival and possibly the N_2_ fixation activities of diazotrophic cyanobacteria (Sañudo-Wilhelmy et al., 2001; Sohm et al., 2011). Nonetheless, these versatile microorganisms may have several adaptive mechanisms to changes in their dynamic marine environment (e.g., nutrient availability) (Tandeau de Marsac and Houmard, 1993; Schwarz and Forchhammer, 2005; Herrero and Flores, 2008).

Phosphorus, (i.e., inorganic phosphorus, ${PO}_{4}^{3-}$), together with iron (Fe) are hypothesized to be the major limiting nutrients for N_2_ fixation (Mills et al., 2004; Moore et al., 2009, 2013). Phosphorus is vital for the storage and retrieval system of genetic information (DNA/RNA), for the energy metabolism through ATP dependence (Kornberg, 1995; Santos-Beneit, 2015; Tiwari et al., 2015), and in most bacteria, it is important for the structure of the cell membrane. During P-starvation, microorganisms produce enzymes that are hydrolyze P-esters contained in dissolved organic phosphorus (DOP) releasing dissolved inorganic phosphorus (DIP), that the cells can utilize. These enzymes are called alkaline phosphatases (APases) and in marine bacteria they are included in three main families: PhoA, PhoX, and PhoD. APases are metalloenzymes that require metal co-factors. PhoA forms a coordinate with two zinc (Zn^2+^) and one magnesium (Mg^2+^) ions; PhoX forms a coordinate with three calcium (Ca^2+^) and one/two Fe^3+^ ions (Yong et al., 2014); and PhoD coordinates with an unknown number of Ca^2+^ ions. In *Bacillus subtilis* model, PhoD has an active site formed with one Fe^3+^ and two Ca^2+^ ions (Rodriguez et al., 2014). This information suggests the possible interaction between metals (e.g., Fe^3+^, Ca^2+^, Mg^2+^, and Zn^2+^) in the mechanisms of P-acquisition involving APases. In *Halothece* sp. PCC 7418, two types of APases have been reported: PhoA (two copies) and PhoD (one copy). Calcium dependence was proven in PhoD in *Halothece* sp. PCC 7418 (Kageyama et al., 2011). However, Fe dependence of PhoD and the relative importance between these two types of APases (PhoA and PhoD) have not been demonstrated in *Halothece* sp. PCC 7418.

APases are included in what is known as the Pho regulon. It is a huge regulatory group of genes that control P-acquisition. Pho regulon is composed of elements related with (1) high-affinity phosphate transport (PstS, PstC, PstA, and PstB) and low-affinity phosphate transport, (2) extracellular enzymes capable of obtaining ${PO}_{4}^{3-}$ from organic phosphates (APases), and (3) polyphosphate metabolism (PpK, PpX, and PpA) as P reservoir or elements with unknown functions (PhoU) (Blanco et al., 2002; Yuan et al., 2006; Santos-Beneit, 2015). PhoU coordinates with metal cluster (Zn^2+^ or Fe^3+^), and may have a role in the control of autokinase activity of the PhoR and Pst systems (Gardner et al., 2014). The Pho regulon is mainly controlled by PhoR-PhoB, a two-component regulatory system (Santos-Beneit, 2015). PhoR is an inner-membrane histidine kinase, while PhoB is a transcriptional factor that recognizes and binds to consensus sequence named PHO box. In cyanobacteria, PHO box is formed by three tandem repeats of 8 bp separated by 5 bp, unlike PHO Box from *Escherichia coli*, formed by two direct repeats of 7 bp separated by 5 bp (Yuan et al., 2006; Su et al., 2007; Tiwari et al., 2015).

The P-acquisition mechanisms in bacteria are well studied in the Atlantic ocean, where Fe is shown to enhance the P-acquisition mechanisms in N_2_-fixing cyanobacterial species, *Trichodesmium* spp. and *Crocosphaera watsonii* (Fu et al., 2005; Dyhrman and Haley, 2006; Browning et al., 2017). However, there is scarcely any information about the relation between metals (e.g., Fe) and P-acquisition mechanisms in N_2_-fixing cyanobacteria found in association with the Mediterranean seagrass, *P*. *oceanica*, taking into account the multiple ecological benefits of this seagrass in the region. The Mediterranean Sea is oligotrophic, characterized by low water column ${PO}_{4}^{3-}$ concentrations and a decreasing gradient of ${PO}_{4}^{3-}$ concentrations from west to east basins (Tanhua et al., 2013). Knowledge on the P-acquisition mechanisms of N_2_-fixing organisms in an environment with limiting levels of ${PO}_{4}^{3-}$ is particularly important. Moreover, the Mediterranean Sea is subject to Saharan atmospheric dust deposition containing Fe (Statham and Hart, 2005), which can play a role in the P-acquisition mechanisms of the organisms.

To study, for the first time, the P-acquisition mechanisms in N_2_-fixing cyanobacteria associated with the dominant coastal ecosystem in the region (*P*. *oceanica* seagrass beds), we selected a diazotrophic unicellular cyanobacteria, *Halothece* sp. found on the leaves of *P*. *oceanica* (Agawin et al., 2017) as our test species. The most related culturable strain is *Halothece* sp. PCC 7418, and this was used as the test organism in this study. The halotolerant *Halothece* sp. PCC 7418 (originally called *Synechococcus* PCC 7418), also known as *Aphanothece halophytica*, was originally isolated from Solar Lake on the eastern shore of the Sinai Peninsula in 1972 (UniProt source). First, we made a genomic analyses of the Pho regulon to check the regulatory group of genes that control the P-acquisition mechanisms and then a structural analysis of PhoD (alkaline phosphatase type D) and PhoU (phosphate acquisition regulator) of *Halothece* sp. PCC 7418 to investigate the connection among metals (e.g., Ca^2+^ and Fe^3+^) and the P-acquisition mechanisms of this species. Second, we experimentally investigated how the availability of Fe affects the alkaline phosphatase activity (APA), their ${PO}_{4}^{3-}$-uptake rates, and the magnitude of the effect under different levels of ${PO}_{4}^{3-}$ and ${\mathrm{NO}}_{3}^{-}$ availability, and how the availability of ${PO}_{4}^{3-}$ and Fe affect Fe-uptake rates of the cells.

## Materials and Methods

### Genome Analysis

With the goal of comparing *Halothece* sp. PCC 7418 Pho regulon with its closest genomes (Luo et al., 2009), the distribution of the number of copies of Pho regulon components in selected strains was analyzed. The genome from *Halothece* sp. PCC 7418 (GenBank: NC_019779.1) and genomes from other closely related microorganisms were compared using the dedicated bacterial information system Pathosystems Resource Integration Center (PATRIC). This database, and the analysis tools included, offers an easy interface in which annotated genes that are included in different subsystems can be searched (Wattam et al., 2017).

### Three-Dimensional Predicted Structures

Sequences of PhoD and PhoU in FASTA format were sent to the I-Tasser server for protein 3D-structure prediction (Zhang, 2008), with their domains previously checked in Pfam 32.0 (Finn et al., 2016). The predicted structures for PhoD and PhoU of *Halothece* sp. PCC 7418 were sent to POSA (Li et al., 2014) for a structural alignment against PhoD of *B*. *subtilis* (PDB: 2YEQ) and PhoU of *Pseudomonas aeruginosa* (PDB: 4Q25), respectively; the two more evolutionarily related homologous proteins available to date in databases (i.e., that have both similar sequences and 3D models). To describe the Fe coordination positions of these proteins, residues from both (i.e., PhoD and PhoU of *Halothece* sp. PCC 7418 against 2YEQ and 4Q25, respectively) were mapped through alignment with Uniprot Clustal Omega (The UniProt Consortium, 2014). The predicted structures and the corresponding structural alignments were visualized with Pymol (DeLano, 2002).

### Strain and Culture Conditions

*Halothece* sp. PCC 7418, was obtained from the Pasteur Culture Collection of Cyanobacteria (PCC) and maintained in 250 ml acid-cleaned Quartz Erlenmeyer flasks containing 150 ml of ASNIII + Tu4X medium (initial pH 7.5) (Stanier et al., 1979). The medium was supplemented with 0.1–0.3% (w/v) of glucose and grown in a rotary shaker (120 r.p.m.) with a photoperiod of 12 h light:12 h dark under low intensity fluorescent light (30 μE m^−2^ s^−1^) at 25°C. Three conditions were established for inorganic phosphorus (${PO}_{4}^{3-}$) concentrations: [Low ${PO}_{4}^{3-}$] (0.1 μM), [Medium ${PO}_{4}^{3-}$] (1 μM), and [High ${PO}_{4}^{3-}$] (45 μM). Furthermore, three conditions for Fe were established: [Low Fe] (2 nM), [Medium Fe] (20 nM), and [High Fe] (7.5 μM). These ${PO}_{4}^{3-}$ and Fe concentration were combined in nine conditions ([Low ${PO}_{4}^{3-}$–Low Fe], [Low ${PO}_{4}^{3-}$–Medium Fe], [Low ${PO}_{4}^{3-}$–High Fe], [Medium ${PO}_{4}^{3-}$–Low Fe], [Medium ${PO}_{4}^{3-}$–Medium Fe], [Medium ${PO}_{4}^{3-}$–High Fe], [High ${PO}_{4}^{3-}$–Low Fe], [High ${PO}_{4}^{3-}$–Medium Fe], and [High ${PO}_{4}^{3-}$–High Fe]), and these treatments were tested in two sets of experiments: growth under 4.4 mM of ${\mathrm{NO}}_{3}^{-}$ (optimal concentration) and 0.15 mM of ${\mathrm{NO}}_{3}^{-}$ (low concentration, and referred from now on as [Low ${\mathrm{NO}}_{3}^{-}$]) (Table 1). The solutions of ${PO}_{4}^{3-}$, Fe, and ${\mathrm{NO}}_{3}^{-}$ were prepared from K_2_HPO_4_, ferric citrate, and NaNO_3_, respectively. The batch cultures were maintained for over 10 days for each experiment and the initial inoculum of cells was added at exponential phase (O.D_750 nm_ ≅ 0.2) from their original ASNIII + Tu4X medium.

**Table 1:** List of all experimental treatments conducted in this study.

Experiments			
Condition (optimal and low $NO_{3}^{-}$)			Description
[Low ${PO}_{4}^{3-}$–Low Fe]	[Medium ${PO}_{4}^{3-}$–Low Fe]	[High ${PO}_{4}^{3-}$–Low Fe]	First experiment – optimal ${\mathrm{NO}}_{3}^{-}$ (4.4 mM)
[Low ${PO}_{4}^{3-}$–Medium Fe]	[Medium ${PO}_{4}^{3-}$–Medium Fe]	[High ${PO}_{4}^{3-}$–Medium Fe]	Second experiment – low ${\mathrm{NO}}_{3}^{-}$ (0.15 mM)
[Low ${PO}_{4}^{3-}$–High Fe]	[Medium ${PO}_{4}^{3-}$–High Fe]	[High ${PO}_{4}^{3-}$–High Fe]	Third experiment – ${\mathrm{NO}}_{3}^{-}$ starvation (6 nM), comparing with optimal ${\mathrm{NO}}_{3}^{-}$ in selected treatments
**Recovery experiments**			
**Initial treatment**	**Condition of** $NO_{3}^{-}$	**Nutrient added (at day 12)**	**Resulting treatment (maintained for 4 days)**
[Low ${PO}_{4}^{3-}$–Low Fe]	Optimal ${\mathrm{NO}}_{3}^{-}$ (4.4 mM)	${PO}_{4}^{3-}$ and Fe	[High ${PO}_{4}^{3-}$–High Fe] in optimal ${\mathrm{NO}}_{3}^{-}$ treatment.
[Low ${PO}_{4}^{3-}$–Low Fe]	${\mathrm{NO}}_{3}^{-}$ starvation (6.66 nM)	${PO}_{4}^{3-}$, Fe, and ${\mathrm{NO}}_{3}^{-}$	[High ${PO}_{4}^{3-}$–High Fe] in optimal ${\mathrm{NO}}_{3}^{-}$ treatment.

*In the recovery experiments,* $PO_{4}^{3-}$*, Fe, and/or* ${NO}_{3}^{-}$ *were added to the different initial treatments to achieve optimal conditions to evaluate the changes in APA rates*.

Selected treatments ([Low ${PO}_{4}^{3-}$–Low Fe], [Low ${PO}_{4}^{3-}$–High Fe], [High ${PO}_{4}^{3-}$–Low Fe], and [High ${PO}_{4}^{3-}$–High Fe]), were also used to compare the results under ${\mathrm{NO}}_{3}^{-}$ starvation (6.66 nM) and optimal ${\mathrm{NO}}_{3}^{-}$ conditions. Cultures were maintained at the same conditions as described above for over 12 days. During the last day, ${PO}_{4}^{3-}$, Fe, and/or ${\mathrm{NO}}_{3}^{-}$ were added to the different treatments to achieve optimal concentrations of ${PO}_{4}^{3-}$ (45 μM), Fe (7.5 μM), and ${\mathrm{NO}}_{3}^{-}$ (4.4 mM) to evaluate the changes in the APA rates, and the new conditions were maintained for over 4 days. The different conditions of the experiments are shown in Table 1.

The importance of PhoD in *Halothece* sp. PCC 7418 was investigated by changing the availability of the metal co-factors for PhoA (Zn^2+^ and Mg^2+^). The method used was as described above in the initial main experiments except that the medium was depleted with Mg and Zn and the condition of ${PO}_{4}^{3-}$ and Fe was: [Medium ${PO}_{4}^{3-}$–High Fe] under optimal ${\mathrm{NO}}_{3}^{-}$.

All cultures were performed in duplicate, and the study parameters (APA, N_2_ fixation, uptake rates of ${PO}_{4}^{3-}$ and Fe, TDP and/or P/Fe/Mn cellular content) were evaluated during the different phases of the culture (O.D_750 nm_ ≅ 0.01–0.2). A subsample of the cells (1.5 ml) was taken from the culture flasks during the experiment and were counted through flow cytometric analysis (as described below) to normalize the results per cell. All samples were manipulated in a class-100 clean hood, to avoid Fe contamination.

### Flow Cytometry Analysis

Cells were fixed with glutaraldehyde 25% (v/v) in H_2_O (Sigma-Aldrich) [final concentration 0.05% (v/v)] and were counted with a Becton Dickinson FACS-Verse cytometer (Beckton & Dickinson, Franklin Lakes, New Jersey, USA). Fluorescent beads, BD FACSuite™ CS&T research beads (Beckton & Dickinson and Company BD Biosciences, San Jose, USA), were used as internal standard to calibrate the instrument. The cytometer shows fluorescence patterns for FITC, PE, PerCP-CyTM5.5 and APC. To count the *Halothece* sp. PCC 7418 cells, we selected FITC (488 nm excitation, 530/30 nm emission) and PE (488 nm excitation, 576/26 nm emission) combination fluorescence signals which show clearly the population of the cells. A total of 10,000 cells were counted in each sample and the counted cells were expressed as cells μl^−1^.

### Alkaline Phosphatase Activity

Alkaline phosphatase activity (APA) was evaluated through a fluorometric assay, in which the hydrolysis of the fluorogenic substrate (S) 4-methylumbelliferyl phosphate (MUF-P, Sigma-Aldrich) to 4-methylumbelliferyl (MUF) was measured. Generally, an end point enzymatic assay was conducted with a concentration of 2 μM MUF-P during the exponential phase of the culture (O.D_750 nm_ ≅ 0.1). After 1 h incubation in darkness at room temperature, APA was measured in a microtiter plate that contained borate buffer at pH 10 (3:1 of sample:buffer). The MUF production (fmole MUF cell^−1^ h^−1^) was measured with a Cary Eclipse spectrofluorometer (FL0902M009, Agilent Technologies) at 359 nm (excitation) and 449 nm (emission) and using a calibration standard curve with commercial MUF (Sigma-Aldrich).

Saturation curves of velocity (V, fmole MUF cell^−1^ h^−1^) vs. substrate (S, μM) were made under [Low ${\mathrm{NO}}_{3}^{-}$] condition during the final exponential phase of the culture (O.D_750 nm_ ≅ 0.2), using different concentrations of MUF-P: 0, 0.05, 0.1, 0.5, 2, and 5 μM. The maximum velocity (Vmax) at saturating substrate concentrations was obtained from each plot of V vs. S. The Michaelis–Menten constant, Km (μM), which represents the substrate concentration at half Vmax was calculated using de Hill plot equation (Ascenzi and Amiconi, 1987). The evolution of MUF-P hydrolysis rates (fmole MUF cell^−1^) with time (h) was recorded over 1 h in the treatments under [Low ${PO}_{4}^{3-}$–Low Fe], [Low ${PO}_{4}^{3-}$–High Fe], and [High ${PO}_{4}^{3-}$–High Fe] at the last day of the experiment with 5 μM of MUF-P under ${\mathrm{NO}}_{3}^{-}$ starvation and ${\mathrm{NO}}_{3}^{-}$ optimal conditions and the APA rate (fmole MUF cell^−1^ h^−1^) was calculated as the slope of the fitted line.

### $PO_{4}^{3-}$ Uptake Rates, Nutrient Concentrations in the Culture Medium and in the Cells

Samples for the determination of ${PO}_{4}^{3-}$ and total dissolved P (TDP) were centrifuged for 15 min at 16,000 ×*g* under 4°C. The supernatant was collected from the centrifuged tubes and used for ${PO}_{4}^{3-}$ determinations following standard spectrophotometric methods (Hansen and Koroleff, 2007). TDP concentrations were also analyzed using the latter method after persulfate digestion. Samples for Fe analyses of culture media were filtered through sterile 0.2 μm filters (MFV5-025, FilterLab) at different times (initial and final) during the experiments. The metal (Fe) concentrations of culture medium were measured by inductively coupled plasma mass spectrometry (ICP-MS; iCap, Thermo Scientific), following the trace-metal clean techniques described in Tovar-Sanchez et al. (2006) and Tovar-Sanchez and Sañudo-Wilhelmy (2011).

The ${PO}_{4}^{3-}$ concentrations in the culture medium were determined at different times: 0, 1, 4, and 10 days in the experimental treatment of [High ${PO}_{4}^{3-}$]: [Low Fe], [Medium Fe], and [High Fe]), and under [Low ${\mathrm{NO}}_{3}^{-}$] and optimal ${\mathrm{NO}}_{3}^{-}$ conditions. Specific ${PO}_{4}^{3-}$ uptake rates (pmole ${PO}_{4}^{3-}$ cell^−1^ day^−1^) were calculated as described in (Ghaffar et al., 2017). Briefly, specific ${PO}_{4}^{3-}$ uptake rates were calculated as the mass balance of ${PO}_{4}^{3-}$over the multiple days by taking the differences of ${PO}_{4}^{3-}$ concentrations at two different times (T_0_–T_1_, T_0_–T_4_, and T_0_–T_10_) and normalized by the number of cells counted at different time points (0, 1, 4, and 10) through the following equation:

(1) $${PO}_{4}^{3-}-\mathrm{uptake}\;\left(\mathrm{pmole}\;{PO}_{4}^{3-}\;{\mathrm{cell}}^{-1}\;{\mathrm{day}}^{-1}\right)=\frac{A-B}{Ti-Tf}$$

*A* is μmole ${PO}_{4}^{3-}$ cell^−1^ at the initial time (*Ti*) and *B* is the μmole ${PO}_{4}^{3-}$ cell^−1^ at the final time (*Tf*).

TDP concentrations were also measured at different times: 0, 4, 8, and 12 days in the experiments under ${\mathrm{NO}}_{3}^{-}$ starvation at [Low ${PO}_{4}^{3-}$] and [High ${PO}_{4}^{3-}$] conditions. Fe-uptake rates were measured under N_2_-fixing conditions (i.e., [Low ${\mathrm{NO}}_{3}^{-}$] conditions). Initial and final Fe concentrations of the culture media were measured, and the difference between time = 0 and time = 10 (T_0_–T_10_) was used to determine the Fe-uptake during the 10 days of the experiment. Specific Fe-uptake (fmole Fe cell^−1^ day^−1^) was calculated the same way as the specific ${PO}_{4}^{3-}$-uptake rates described above.

Cellular contents of phosphorus (P), Fe, and other metals (i.e., Mn, V, Co, Ni, or Zn) were also determined by collecting the cells under [Low ${\mathrm{NO}}_{3}^{-}$] treatment conditions through filtration of a known volume of culture (20 ml) with 0.2-μm acid-cleaned polycarbonate filters (Merck-Millipore). Elemental concentrations of P and Fe in the cyanobacterial samples were determined by inductively coupled plasma mass spectrometry (ICP-MS; iCap, Thermo Scientific), after microwave acid digestion (CEM, Mars 5) using nitric acid (high purity Suprapur^®^, Merck) (Tovar-Sanchez et al., 2006; Tovar-Sanchez and Sañudo-Wilhelmy, 2011).

### Acetylene Reduction Assay

N_2_-fixing activities were measured with the acetylene reduction assay (ARA) method under known N_2_-fixing conditions for unicellular cyanobacteria (i.e., low ${\mathrm{NO}}_{3}^{-}$ concentrations, anaerobic environment, dark phase of the photoperiod, Reddy et al., 1993), and under low-medium levels of Fe and in low-medium-high levels of ${PO}_{4}^{3-}$. A volume of 50 ml from treatments with [Low ${\mathrm{NO}}_{3}^{-}$] condition at day 8 of the experiment was transferred to anaerobic tubes for cultivation for 2 days, and after which, ARA measurements were done following the method described in Agawin et al. (2014). Duplicate 10 ml samples of culture from each experimental tube, were filtered through 0.45 μm GF/F filters (MFV5-025, FilterLab). The filters were deposited in hermetic vials containing 1 ml of the corresponding culture medium. Acetylene (C_2_H_2_) was added at 20% (v/v) final concentration in each vial using gas-tight Hamilton syringes. The filters were incubated in the vials for 3 h at room temperature in the dark. After 3 h incubation time, 10 ml of headspace gas were removed with a gas-tight Hamilton syringe from the incubation vials or tubes, transferred and stored in Hungate tubes and sealed with hot melt adhesive glue (SALKI, ref. 0430308) to minimize gas losses (Agawin et al., 2014). Ethylene and acetylene were determined using a GC (model HP-5890, Agilent Technologies) equipped with a flame ionization detector (FID). The column was a Varian wide-bore column (ref. CP7584) packed with CP-PoraPLOT U (27.5 m length, 0.53 mm inside diameter, 0.70 mm outside diameter, and 20 μm film thickness). Helium was used as carrier gas at a flow rate of 30 ml min^−1^. Hydrogen and airflow rates were set at 30 and 365 ml min^−1^, respectively. The split flow was used so that the carrier gas flow through the column was 4 ml min^−1^ at a pressure of 5 psi. Oven, injection, and detector temperatures were set at 52, 120, and 170°C, respectively. Ethylene produced was calculated using the equations in Stal (1988). The acetylene reduction rates were converted to N_2_ fixation rates (pmole N_2_ ml^−1^ h^−1^) using a factor of 4:1 (Jensen and Cox, 1983).

### Statistical Analyses

Univariate Analysis of variance (ANOVA) factor analyses and *post-hoc* (Bonferroni) was used to study the effect of the nutrient treatment conditions to APA rates, P-cellular content and specific ${PO}_{4}^{3-}$, and Fe uptake rates. In other cases, where we want to highlight a specific point, we use individual *t* tests. Regression analyses were used to determine the relationships between P-cellular content vs. N_2_ rates fixation, P-cellular content vs. Fe-cellular content and P/Fe-cellular content vs. other metals (i.e., Mn). The statistical analyses were performed using the SPSS program version 21 (IBM Corp year 2012).

## Results

### Pho Regulon of *Halothece* sp. PCC 7418

**Figure 1:**
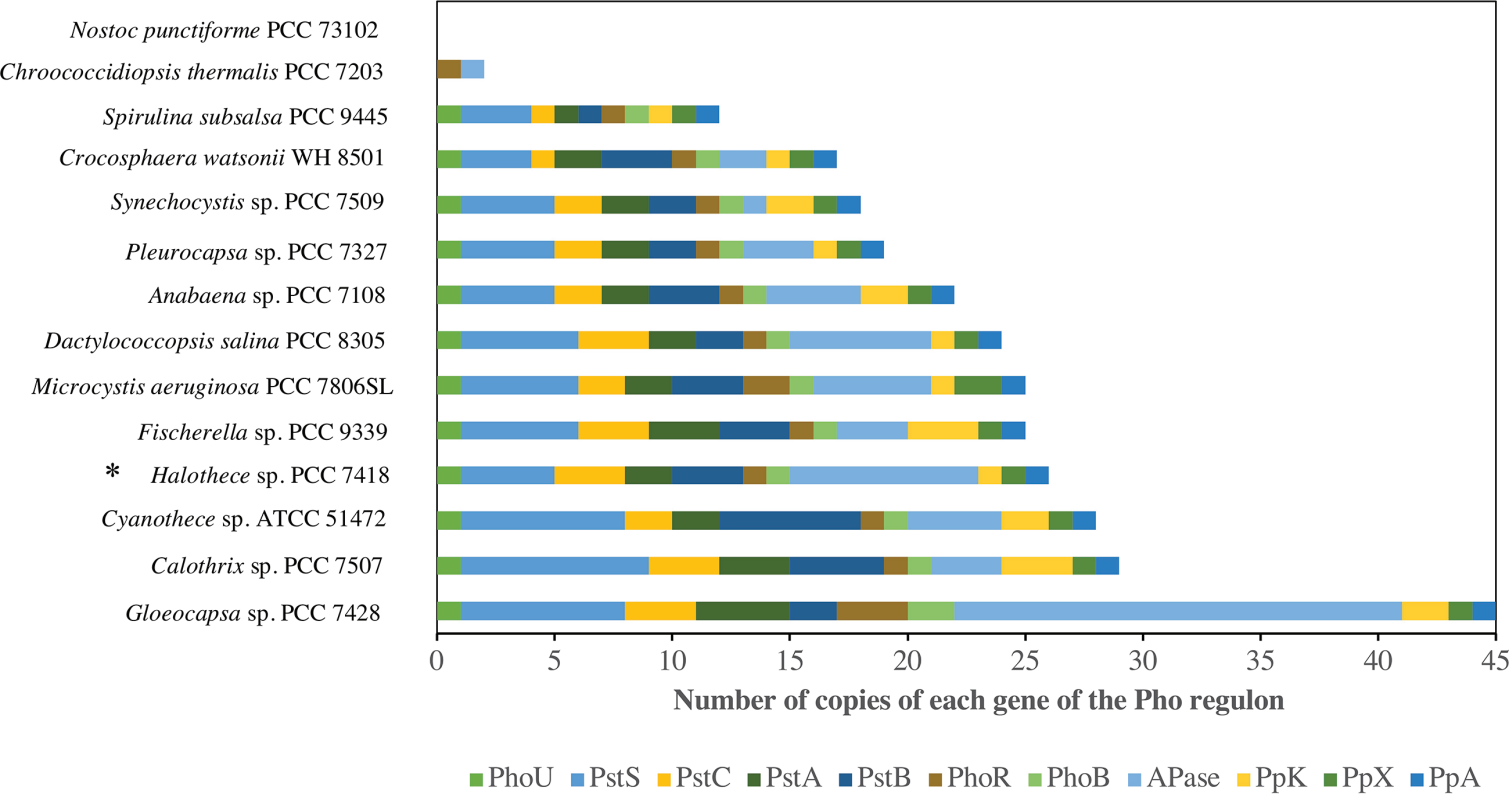
Distribution of the Pho regulon in *Halothece* sp. PCC 7418 (*) and genomes of its closest cyanobacterial relatives. Annotated genes were identified in PATRIC.

The distribution of the number of copies of Pho regulon components of *Halothece* sp. PCC 7418 and its closest genomes (Luo et al., 2009) are shown in Figure 1 and Supplementary Table S1. The *Gloeocapsa* sp. PCC 7428 genome had the highest number of copies detected (up to 45), suggesting that this species is one of the better adapted species to P-limitation. On the other hand, *Nostoc punctiforme* PCC 73102 and *Chroococcidiopsis thermalis* PCC 7203 genomes had the lowest number of copies of the Pho regulon components. Our test microorganism *Halothece* sp. PCC 7418 genome was the fourth cyanobacterium containing more copies of the Pho regulon (26): 1 for *phoU*, 4 for *pstS*, 3 for *pstC*, 2 for *pstA*, 3 for *pstB*, 1 for *phoR*-*phoB*, 8 for APases, 1 for *ppK*, 1 for *ppX,* and 1 for *ppA*. With eight copies of APases, it was the second cyanobacterium containing more APases (8), only surpassed by *Gloeocapsa* sp. PCC 7428 (19), suggesting a key role of the APases in *Halothece* sp. PCC 7418. Annotation in PATRIC did not annotate any specific APase, except for a PhoD. No low-affinity phosphate transporters were detected.

### Structural Analysis of PhoD and PhoU of *Halothece* sp. PCC 7418

#### Three-Dimensional Structure of PhoD and Its Implication in Alkaline Phosphatase Activity

**Figure 2:**
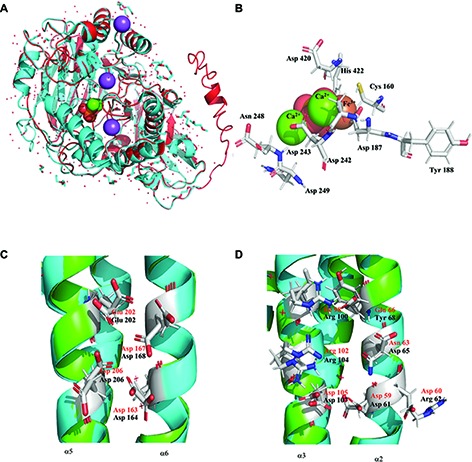
Structural analysis of PhoD and PhoU of *Halothece* sp. PCC 7418. **(A)** Predicted structure of PhoD of *Halothece* sp. PCC 7418 is represented in red and aligned with the crystal structure 2YEQ that is displayed in blue cyan. **(B)** Active center of PhoD of the test bacterium showing all the aminoacids involved in coordination with two Ca^2+^ and Fe^3+^ ions. **(C)** Cluster 1 of PhoU, trinuclear metal site with Fe, between α5 and α6. **(D)** Cluster 2 of PhoU, tetranuclear metal site with three Fe and one Ni, between α2 and α3. Black amino acids are from predicted PhoU and red amino acids are from 4Q25. All the structures were represented with Pymol.

The annotated PhoD of *Halothece* sp. PCC 7418 displayed 511 amino acids (aa) with two domains i.e., PhoD-like phosphatase N-terminal domain and PhoD-like phosphatase domain. Predicted structure of PhoD (C-score = 0.00, estimated TM-score = 0.71 ± 0.11, estimated RMSD = 7.4 ± 4.3 Å) had 10 α-helix and 21 β-chains. PhoD of *Halothece* sp. PCC 7418 was homologue to the crystal structure of PhoD of *B*. *subtilis* (2YEQ) of 522 aa, with an identity of 47.5% and coverage of 91.6%. Figure 2A shows the structural alignment between PhoD of *Halothece* sp. PCC 7418 and 2YEQ. The sequence alignment displayed up to 40.11% similarity and was used with the intention to describe the hypothetical catalytic center. The catalytic center for PhoD of *Halothece* sp. PCC 7418, using the catalytic center of 2YEQ (in parenthesis) as a template, consisted of Cys 160 (Cys 124), Asp 187 (Asp 151), Tyr 188 (Tyr 152), Asp 242 (Asp 209), Asp 243 (Asp 210), Asn 248 (Asn 215), Asp 249 (Asn 216), Asp 420 (Asp 380), and His 422 (His 382) (Figure 2B). All these amino acids are described in 2YEQ as the active site and coordinate with two Ca^2+^ and one Fe^3+^ ions (Rodriguez et al., 2014). Only one substitution was detected in Asp 249, where in 2YEQ is Asn 216.

**Figure 3:**
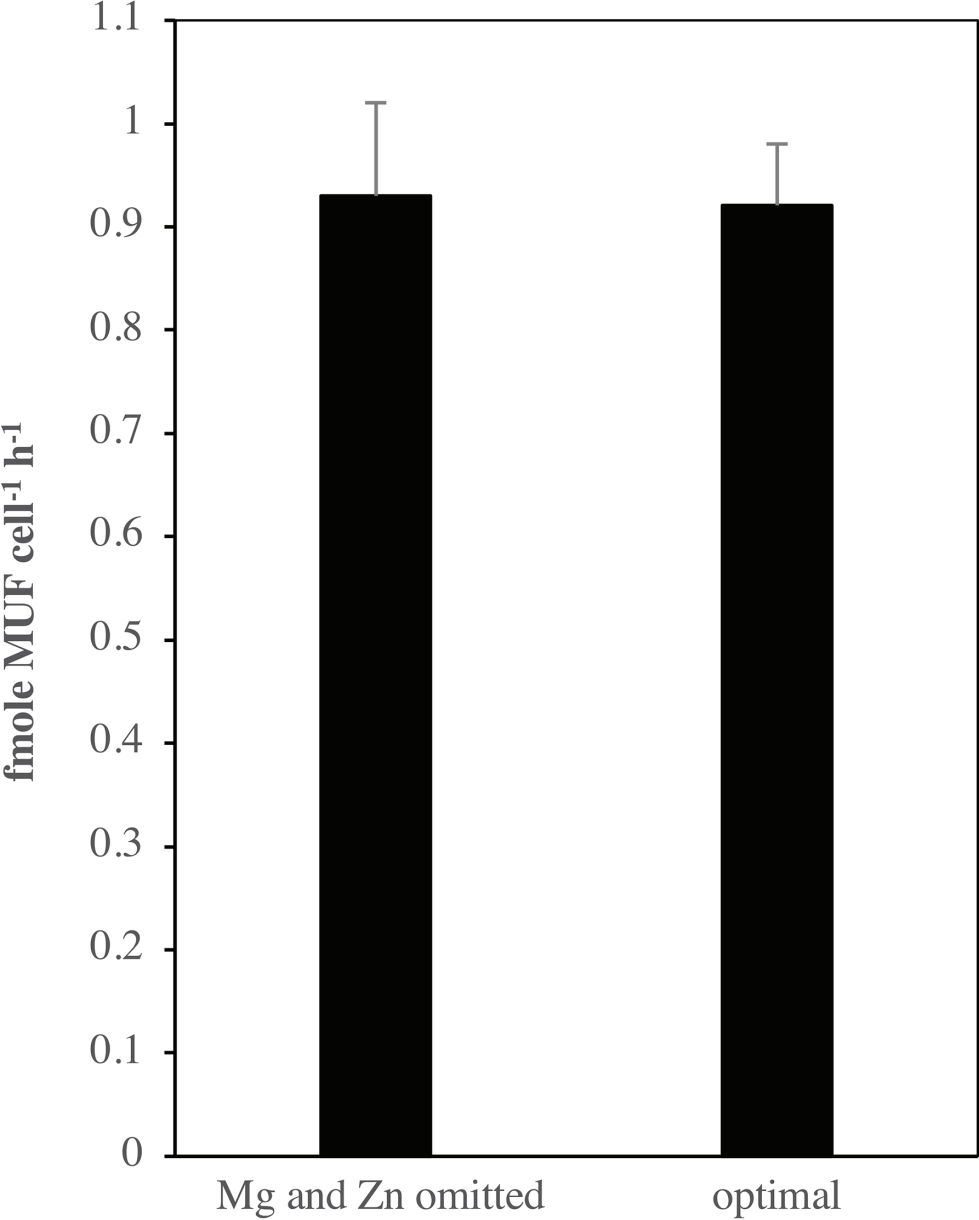
End point assay measurement of alkaline phosphatase activity (APA) under Mg/Zn omission compared with optimal condition. Values are the mean, and the error bar is the spanning range between the two duplicate measurements.

The *in-silico* results described above of PhoD and how it coordinates with Ca^2+^ and Fe^3+^ ions in its active site in *Halothece* sp. PCC 7418 corroborates with the results of the experiment testing the relative importance of PhoD and PhoA in *Halothece* sp. PCC 7418, showing that the APA rates, with the depletion of Mg^2+^ and Zn^2+^ which are the metal co-factors of PhoA, did not differ considerably with sufficient availability of Mg^2+^ and Zn^2+^ (Figure 3). This suggests that PhoD (and not PhoA) is the more active APase in *Halothece* sp. PCC 7418.

#### Three-Dimensional Structure of PhoU

Annotated PhoU had 224 amino acids (aa) and presented two PhoU domains. The predicted structure of PhoU (C-score = 0.55, estimated TM-score = 0.79 ± 0.09, estimated RMSD = 4.5 ± 2.9 Å) had seven α-helix without β-chains. The protein with more structure homology was PhoU of *P*. *aeruginosa* (4Q25) of 250 aa with an identity of 32.5% and coverage of 93.3%. Sequence alignment with 4Q25 showed 27.45% of identity and we used this alignment to describe its metal clusters (Figures 2C,D). Results showed that *Halothece* sp. PCC 7418 using 4Q25 as a template displayed at least one metal cluster, and possibly a second one, forming a trinuclear metal site with three Fe and tetranuclear metal site with three Fe and one nickel (Ni). The first cluster was complete and had the same aa as *P*. *aeruginosa* (in parenthesis) and was formed by Asp 164 (Asp 163), Asp 168 (Asp 167), Glu 202 (Glu 202), and Asp 206 (Asp 206), between α-helixes 5 and 6 (Figure 2C). The second cluster was incomplete and did not have all the aa that are present in *P*. *aeruginosa*. Only three aa of seven aa in *P*. *aeruginosa* (in parenthesis) coincide with *Halothece* sp. PCC 7418, and this cluster consisted of Asp 61 (Asp 59), Arg 62 (Asp 60), Asp 65 (Asn 63), Tyr 68 (Glu 66), Arg 100 (Ile 98), Arg 104 (Arg 102), and Asp 107 (Asp 105) between α-helix 2 and 3 (Figure 2D; Lee et al., 2014).

### Alkaline Phosphatase Activity in *Halothece* sp. PCC 7418

**Figure 4:**
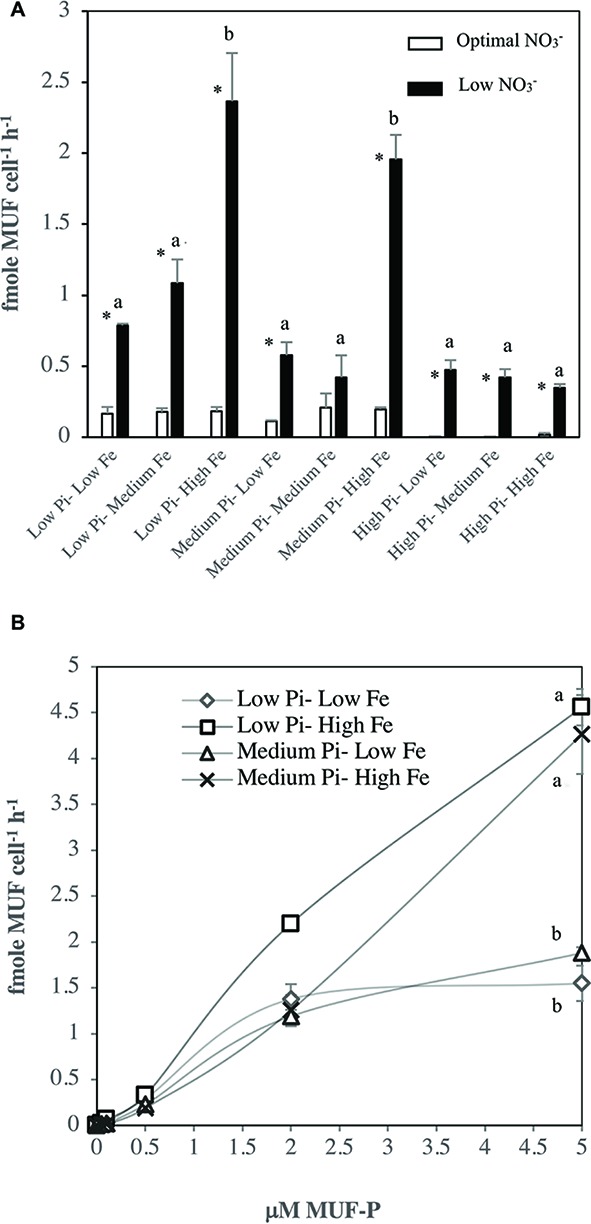
Rates of alkaline phosphatase activity (APA) in *Halothece* sp. PCC 7418 under optimal ${\mathrm{NO}}_{3}^{-}$ and [Low ${\mathrm{NO}}_{3}^{-}$] conditions. **(A)** End point assay of APA under optimal and [Low ${\mathrm{NO}}_{3}^{-}$] conditions with 2 μM of MUF-P. **(B)** Saturation curve, velocity (V, fmole MUF cell^−1^ h^−1^) vs. substrate (S, μM) under [Low ${\mathrm{NO}}_{3}^{-}$] in [Low ${PO}_{4}^{3-}$–Low Fe], [Low ${PO}_{4}^{3-}$–High Fe], [Medium ${PO}_{4}^{3-}$–Medium Fe], and [Medium ${PO}_{4}^{3-}$–High Fe], up to 5 μM of MUF-P. ${PO}_{4}^{3-}$ is represented as Pi. In **(A,B)**, values are the mean and the error bar is the spanning range between the duplicate measurements. Asterisks (*) indicate significant differences (*p* < 0.05) between ${\mathrm{NO}}_{3}^{-}$ optimal and [Low ${\mathrm{NO}}_{3}^{-}$] conditions of the same ${PO}_{4}^{3-}$ and Fe combination treatments, by individual *t* student test. Different letters indicate pairwise significant differences (*p* < 0.05) among treatments in [Low ${\mathrm{NO}}_{3}^{-}$] using a *post-hoc* test (Bonferroni) after ANOVA over the whole dataset was done.

Generally, APA rates were significantly higher (*p* < 0.05) in [Low ${\mathrm{NO}}_{3}^{-}$] conditions compared with optimal ${\mathrm{NO}}_{3}^{-}$ conditions (Figure 4A). Under [Low ${\mathrm{NO}}_{3}^{-}$] APA rates were ≈ 7 times higher in [Low–Medium ${PO}_{4}^{3-}$] and ≈ 77 times higher in [High ${PO}_{4}^{3-}$] compared with their rates under optimal ${\mathrm{NO}}_{3}^{-}$conditions. Moreover, under optimal ${\mathrm{NO}}_{3}^{-}$ conditions, APA rates did not have significant differences among the treatments (Figure 4A). Under [Low ${\mathrm{NO}}_{3}^{-}$], treatment combinations of ${PO}_{4}^{3-}$ and Fe levels had a significant effect on APA rates (ANOVA, *p* < 0.05), where the rates were significant higher (*p* < 0.05) at the highest Fe levels and at low to medium ${PO}_{4}^{3-}$ levels, compared with other treatment combinations (Figure 4A). Figure 4B shows the differences in the kinetics of APA for treatments under [Low ${\mathrm{NO}}_{3}^{-}$] at low and medium ${PO}_{4}^{3-}$ levels and low and high Fe levels. At high Fe levels with low to medium ${PO}_{4}^{3-}$ levels, the V vs. S curve did not reach saturation levels with the maximum S added (5 μM MUF-P). The Vmax and Km, calculated using the available data for these treatments, were: Vmax, 4.92 ± 0.56 fmole cell^−1^ h^−1^; Km of 3.47 ± 0.94 μM at [Low ${PO}_{4}^{3-}$–High Fe] and Vmax, 4.26 ± 0.43 fmole cell^−1^ h^−1^; Km of 7.24 ± 0.57 μM at [Medium ${PO}_{4}^{3-}$–High Fe]. On the contrary to high Fe levels, APase kinetics reached saturation levels with the maximum S added (Figure 4B) at low Fe levels with Vmax, 1.55 ± 0.19 fmole cell^−1^ h^−1^; Km of 1.53 ± 0.31 μM at [Low ${PO}_{4}^{3-}$–Low Fe] and Vmax, 1.88 ± 0.06 fmole cell^−1^ h^−1^; Km of 2.02 ± 0.94 μM at [Medium ${PO}_{4}^{3-}$–Low Fe].

**Figure 5:**
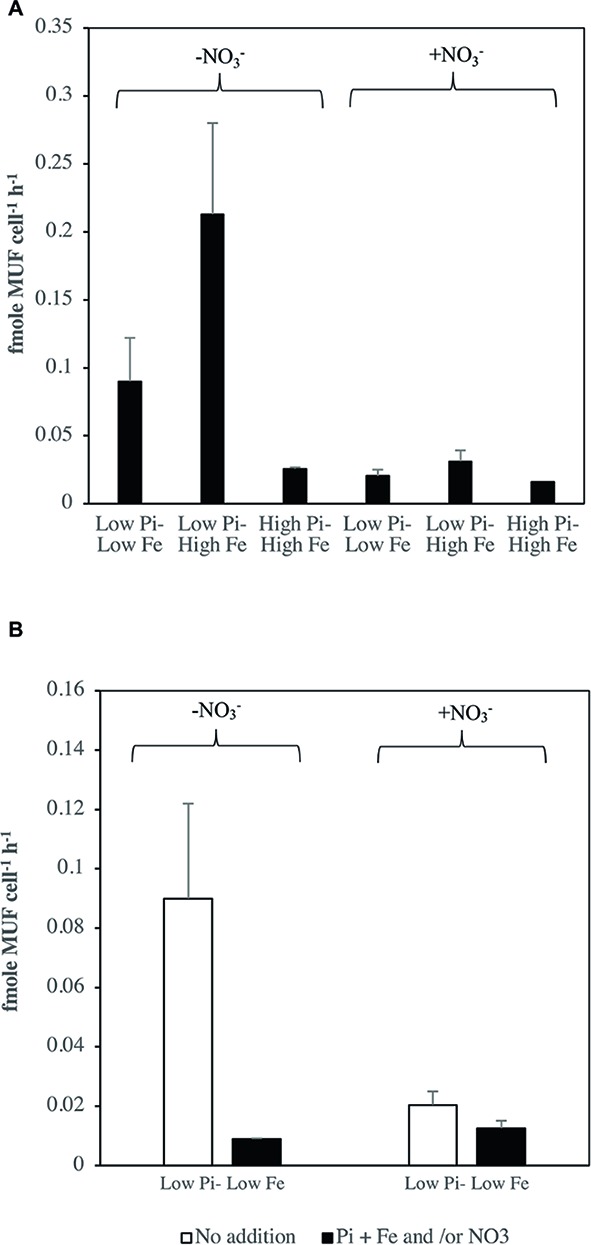
Rates of alkaline phosphatase activity (APA, fmole MUF cell^−1^ h^−1^) in *Halothece* sp. PCC 7418 under ${\mathrm{NO}}_{3}^{-}$ starvation conditions compared with the optimal ${\mathrm{NO}}_{3}^{-}$ treatments under **(A)** treatment conditions: [Low ${PO}_{4}^{3-}$–Low Fe], [Low ${PO}_{4}^{3-}$–High Fe], and [High ${PO}_{4}^{3-}$–High Fe] and **(B)** under re-inoculum of nutrients in treatment conditions: [Low ${PO}_{4}^{3-}$–Low Fe, + ${PO}_{4}^{3-}$ + Fe + ${\mathrm{NO}}_{3}^{-}$] and [Low ${PO}_{4}^{3-}$–Low Fe + ${PO}_{4}^{3-}$ + Fe]. ${PO}_{4}^{3-}$ is represented as Pi. Values are the mean, and the error bar is the spanning range between the duplicate measurements.

The APA rates calculated were considerable higher (up to 6-fold) under ${\mathrm{NO}}_{3}^{-}$ starvation compared with under ${\mathrm{NO}}_{3}^{-}$ optimal conditions (Figure 5A). Treatments with [High Fe] in [Low ${PO}_{4}^{3-}$] reached the maximum rates at 0.21 ± 0.07 fmole cell^−1^ h^−1^ under ${\mathrm{NO}}_{3}^{-}$ starvation condition, and at 0.03 ± 0.01 fmole cell^−1^ h^−1^ under ${\mathrm{NO}}_{3}^{-}$ optimal condition (Figure 5A). Additions of ${PO}_{4}^{3-}$, Fe, and/or ${\mathrm{NO}}_{3}^{-}$ to obtain optimum concentration of ${PO}_{4}^{3-}$, Fe, and/or ${\mathrm{NO}}_{3}^{-}$ resulted in reduced APA rates particularly under the initial ${\mathrm{NO}}_{3}^{-}$ starvation conditions (Figure 5B).

### Phosphorus-Uptake at Different Levels of Iron Availability and Iron-Uptake at Different Levels of $PO_{4}^{3-}$ Availability

**Figure 6:**
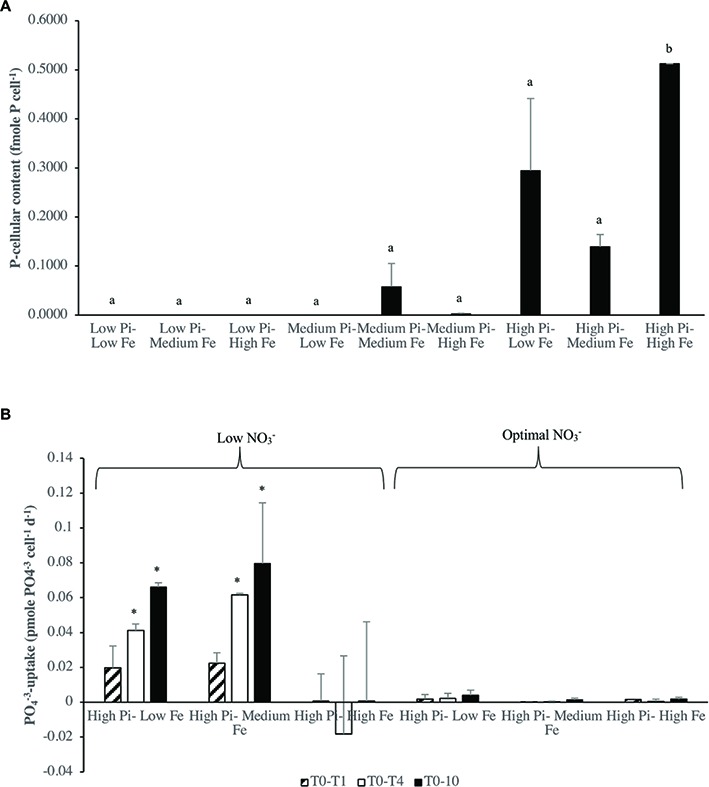
**(A)** P-cellular content (fmole P cell^−^1) under N_2_-fixing conditions in *Halothece* sp. PCC 7418, at different ${PO}_{4}^{3-}$ and Fe levels. **(B)** Specific net ${PO}_{4}^{3-}$-uptake rates (pmole ${PO}_{4}^{3-}$-uptake cell^−1^ day^−1^) in *Halothece* sp. PCC 7418 under [Low ${\mathrm{NO}}_{3}^{-}$] and optimal ${\mathrm{NO}}_{3}^{-}$ conditions at three different periods (T_0_–T_1_, T_0_–T_4_, and T_4_–T_10_). ${PO}_{4}^{3-}$ is represented as Pi. Values are the mean, and the error bar is the spanning range between the duplicate measurements. Different letters indicate pairwise significant differences (*p* < 0.05) among treatments using a *post-hoc* test (Bonferroni) after ANOVA over the whole dataset was done, and asterisks (*) indicate significant differences (*p* < 0.05) between [Low ${\mathrm{NO}}_{3}^{-}$] and ${\mathrm{NO}}_{3}^{-}$ optimal conditions by individual *t* student tests for each treatment combination of ${PO}_{4}^{3-}$ and Fe.

Generally, P-cellular content varied significantly (ANOVA, *p* < 0.05) under N_2_-fixing conditions [Low ${\mathrm{NO}}_{3}^{-}$] among treatment combinations with significantly higher values at [High ${PO}_{4}^{3-}$–High Fe] treatment compared with other treatment combinations (Figure 6A). On the other hand, specific ${PO}_{4}^{3-}$-uptake rates under N_2_-fixing conditions [Low ${\mathrm{NO}}_{3}^{-}$] and optimal ${\mathrm{NO}}_{3}^{-}$ conditions generally did not vary significantly (ANOVA, *p* > 0.05) among treatment combinations (Figure 6B). However, specific *t*-tests conducted under [Low ${\mathrm{NO}}_{3}^{-}$] conditions, showed ${PO}_{4}^{3-}$-uptake rates to be on average 200 times significantly higher (*p* < 0.05) than the rates under optimal conditions of ${\mathrm{NO}}_{3}^{-}$ in T_0_–T_4_ and T_0_–T_10_ in low to medium Fe levels (Figure 6B). Different concentrations of Fe in [High ${PO}_{4}^{3-}$] did not show significant differences in ${PO}_{4}^{3-}$-uptake rates (*p* > 0.05) (Figure 6B).

**Figure 7:**
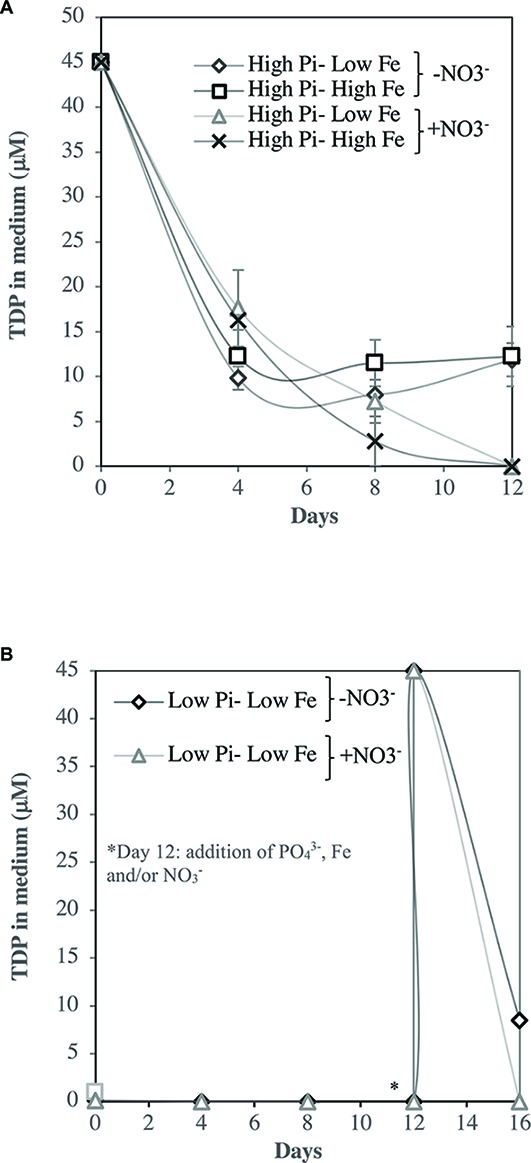
TDP (μM) under ${\mathrm{NO}}_{3}^{-}$ starvation and optimal ${\mathrm{NO}}_{3}^{-}$ conditions. **(A)** TDP consumption (μM) under ${\mathrm{NO}}_{3}^{-}$ starvation and ${\mathrm{NO}}_{3}^{-}$ optimal conditions. **(B)** TDP consumption in the re-inoculated conditions in [Low ${PO}_{4}^{3-}$–Low Fe] (under ${\mathrm{NO}}_{3}^{-}$ starvation and ${\mathrm{NO}}_{3}^{-}$ optimal conditions). ${PO}_{4}^{3-}$ is represented as Pi. Values are the mean, and the error bar is the spanning range between the duplicate measurements. In **(A)**, letters indicate significant differences (*p* < 0.05) by *t* student test.

The time course of depletion of total dissolved phosphate (TDP) in the culture media showed that under optimal ${\mathrm{NO}}_{3}^{-}$ conditions, the media were depleted with TDP while under ${\mathrm{NO}}_{3}^{-}$ starvation conditions, the cells were not capable in depleting TDP from the media (Figure 7A). Fe did not have a significant effect in TDP depletion (*p* > 0.05). The time course of depletion of TDP in the re-inoculum conditions at [Low ${PO}_{4}^{3-}$–Low Fe] (under ${\mathrm{NO}}_{3}^{-}$ starvation and ${\mathrm{NO}}_{3}^{-}$ optimal conditions), showed the same tendency, in which under ${\mathrm{NO}}_{3}^{-}$ starvation conditions, TDP was not depleted (Figure 7B).

**Figure 8:**
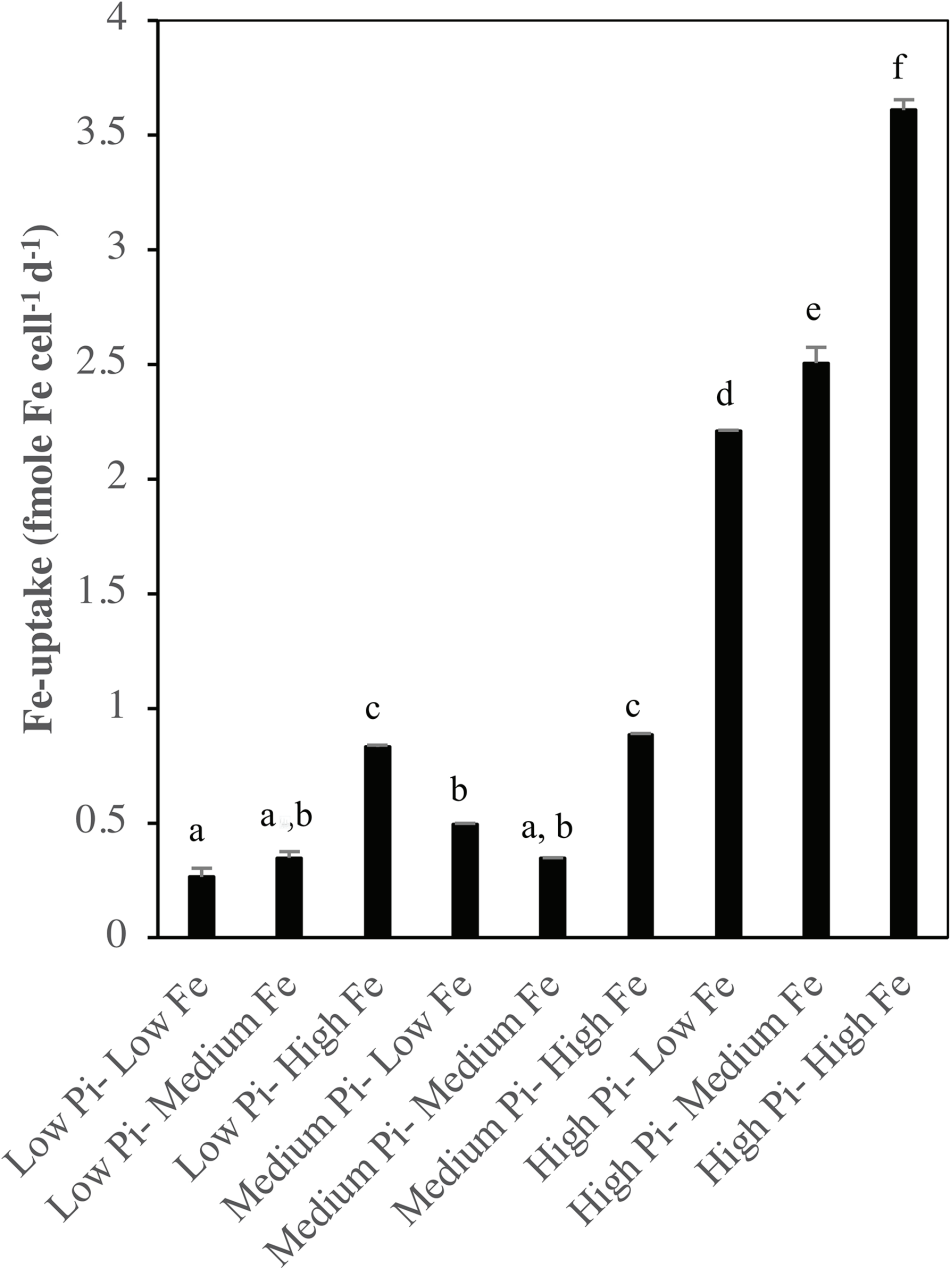
Fe-uptake in *Halothece* sp. PCC 7418 (fmole Fe cell^−1^ day^−1^) measured as the difference of the initial Fe concentration and the final Fe concentration at day 10 day of the experiment under [Low ${\mathrm{NO}}_{3}^{-}$]. ${PO}_{4}^{3-}$ is represented as Pi. Values are the mean, and the error bar is the spanning range between the duplicate measurements, and letters indicate significant differences (*p* < 0.05) between treatments using a *post-hoc* test (Bonferroni) after ANOVA over the whole dataset was done.

Figure 8 shows the specific Fe-uptake rates at different levels of ${PO}_{4}^{3-}$ and Fe under N_2_-fixing conditions. Results showed that generally, specific Fe-uptake rates varied significantly at different treatment combinations of ${PO}_{4}^{3-}$ and Fe (ANOVA, *p* < 0.05). Fe-uptake rates were significantly higher (*p* < 0.05) at [High ${PO}_{4}^{3-}$] conditions compared to [Low ${PO}_{4}^{3-}$] and [Medium ${PO}_{4}^{3-}$] conditions. There were also significant differences (*p* < 0.05) of increased Fe-uptake with increasing availability of Fe.

### Phosphorus-Cellular Content and Its Relationship With N_2_ Fixation and Iron-Cellular Content

**Figure 9:**
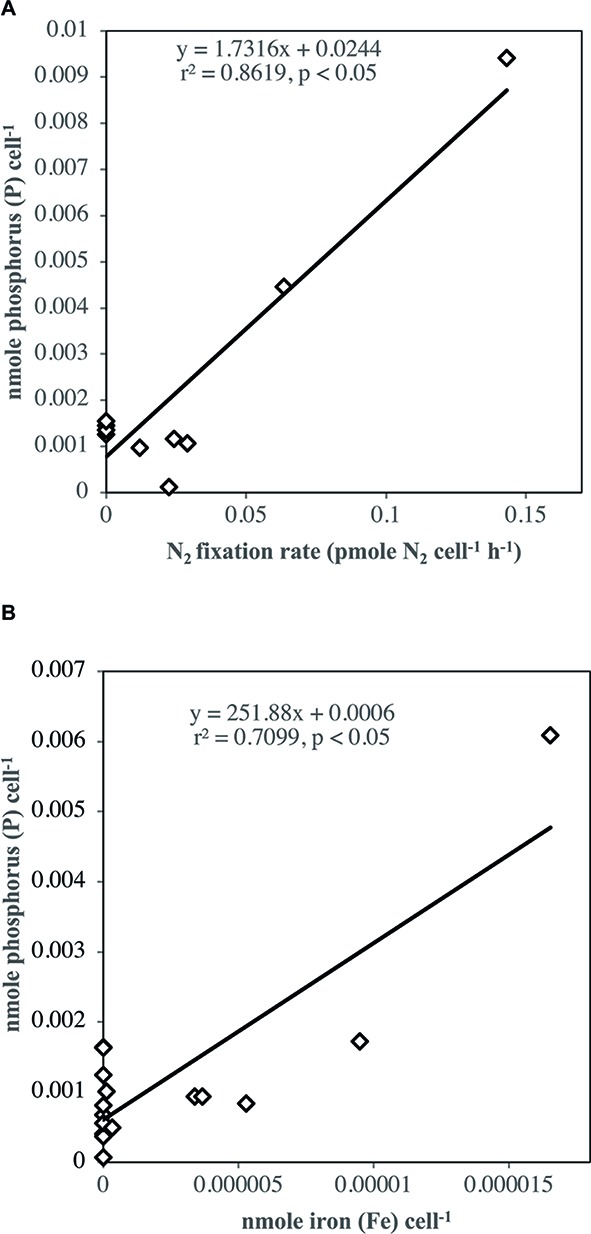
Linear regression analyses **(A)** between P-cellular content and N_2_ fixation and **(B)** between P and Fe-cellular content. Cellular content was measured in [Low ${\mathrm{NO}}_{3}^{-}$] using pooled data from all ${PO}_{4}^{3-}$ and Fe combination treatments.

Phosphorus cellular content of *Halothece* sp. PCC 7418 showed significant positive linear correlation with N_2_ fixation rates (*p* < 0.05, *r*^2^ = 0.86, *n* = 12) (Figure 9A). Moreover, the P-cellular content of the cells showed significant positive linear correlation with their Fe contents (*p* < 0.05, *r*^2^ = 0.71, *n* = 18) (Figure 9B). The P and Fe-cellular contents of the cells did not show significant correlations with other metals (i.e., Mn).

## Discussion

### Pho Regulon and the Three-Dimensional Structure of PhoD and PhoU of *Halothece* sp. PCC 7418: Elucidating the Role of Iron as Co-factor

The Pho regulon of *Halothece* sp. PCC 7418 is composed of genes whose protein products are involved in different functions: autokinase activity of PhoR and phosphate transport (PhoU); high-affinity phosphate transport (PstS, PstC, PstA, and PstB), in a two-component regulatory system (PhoR-PhoB); extracellular enzymes capable of obtaining ${PO}_{4}^{3-}$ from organic phosphates (Alkaline Phosphatases, APases); and polyphosphate metabolism (PpK, PpX, and PpA) (Santos-Beneit, 2015). However, no low-affinity transporters were annotated while some studies demonstrated that this strain exhibited low-affinities transporters (Tripathi et al., 2013). *Halothece* sp. PCC 7418 contains a Pho regulon with 11 distinct genes in single or multiple copies altogether accounting 26 distinct loci in the whole genome, suggesting that *Halothece* sp. PCC 7418 is well adapted to survive to P-limiting conditions. In model strains whose P-acquisition mechanisms are well studied such as *Trichodesmium* spp. and *Crocosphaera watsonii* they only have 15 copies and 19 copies respectively in their Pho regulon (Fu et al., 2005; Dyhrman and Haley, 2006).

Genome analysis indicated that *Halothece* sp. PCC 7418 and *Gloeocapsa* sp. PCC 7428 were the strains with more copies of Alkaline Phosphatases (APase), 8 and 19, respectively (Figure 1). These two cyanobacteria are halotolerant species, and there are studies that suggest that salt stress enhance APA in halophytic strains (Kageyama et al., 2011). In a previous study (Kageyama et al., 2011), *Halothece* sp. PCC 7418 only showed three APases: two PhoA and one PhoD. Of the eight APases found in our study for the same species, one of them is also annotated as PhoD and the rest are not annotated to a specific type of APase. PhoD, together with PhoX, is one of the most abundant APases in marine habitats and its activity may be controlled by availability of its metal co-factor(s) (e.g., Fe^3+^, Ca^2+^, Mg^2+^, and Zn^2+^) (Luo et al., 2009; Zeng et al., 2011).

Three-dimensional analyses with PhoD of *Halothece* sp. PCC 7418 revealed its active site as a homologue to the crystal structure of PhoD of *B*. *subtilis* with two Ca^2+^ and one Fe^3+^ ions as co-factors (Figure 2B; Rodriguez et al., 2014). Previous studies on APase activity in *Halothece* sp. PCC 7418 indicated Ca^2+^ dependence of PhoD (Kageyama et al., 2011) but the Fe^3+^ dependence was not investigated. The experiment conducted here wherein the omission of Mg^2+^ and Zn^2+^ (but not Fe^3+^ in the culture medium) did not result in any significant changes in APase activity (Figure 3), suggesting that the APases of *Halothece* sp. PCC 7418 (i.e., PhoD) do not require these metals (Mg^2+^ and Zn^2+^) as co-factors as in the case of PhoA (Kageyama et al., 2011), and the most active APase could be PhoD.

Iron is not only important as a co-factor for the activities of APase but can be essential in other components of Pho regulon like PhoU in which the results of the 3D-dimensional analyses in this study showed PhoU of *Halothece* sp. PCC 7418 forming at least one Fe-containing metal cluster, and possible a second cluster (Figures 2C,D), using as a model, the PhoU of *P*. *aeruginosa* (4Q25). PhoU can participate in the ${PO}_{4}^{3-}$ transport across the cell membranes of bacteria in the regulation of the phosphate-specific transport systems (Santos-Beneit, 2015) and in controlling cellular phosphate metabolism (Lubin et al., 2015). The specific role of PhoU in *Halothece* sp. PCC 7418, however, remains to be investigated.

### Alkaline Phosphatase Activity in *Halothece* sp. PCC 7418: Experimental Analysis of Regulation by Iron, $NO_{3}^{-}$, and Phosphorous Availabilities

Experimental measurements of APA in *Halothece* sp. PCC 7418 under different levels in Fe availability revealed higher activities with higher levels of Fe (Figures 4A,B and Figure 5A), confirming the regulatory role of Fe in the APase (i.e., PhoD) in this species as we predicted in our 3D-structural analyses of its PhoD (Figure 2B). However, the effect of Fe availability on the rates of APA in *Halothece* sp. PCC 7418 depends on the availability of inorganic sources of nitrogen (i.e., ${\mathrm{NO}}_{3}^{-}$) wherein at low ${\mathrm{NO}}_{3}^{-}$ concentrations, increasing Fe availability enhanced the APA rates (Figures 4A,B). We showed that under [Low ${\mathrm{NO}}_{3}^{-}$] and at high Fe levels, APA was not saturated (Figure 4B). We hypothesized that under these conditions, the Vmax of APases from *Halothece* sp. PCC 7418 is so high that increasing MUF-P concentrations, up to 10 μM (in the other assays that were additionally conducted),was not high enough to saturate the enzyme because of the enhancement of APA by high levels of the Fe co-factor.

At high or optimal ${\mathrm{NO}}_{3}^{-}$ concentrations, APA rates in general are lower than in ${\mathrm{NO}}_{3}^{-}$ starvation conditions (Figure 5A) and even lower than in [Low ${\mathrm{NO}}_{3}^{-}$] treatments (Figure 4A). These results can be due to peculiar characteristics of the N_2_ fixation process. High concentrations of readily assimilable forms of dissolved inorganic nitrogen (DIN, i.e., NH_4_, ${\mathrm{NO}}_{3}^{-}$) are known to inhibit N_2_ fixation as evidenced by DIN inhibition studies (Knapp, 2012). The N_2_ fixation process (N_2_ + 8e^−^ + 16ATP + 8H^+^ → 2NH_3_ + H_2_ + 16ADP + 16${PO}_{4}^{3-}$) is an energetically costly processes requiring 16 ATPs and 25% more energy is needed to reduce N_2_ than to reduce ${\mathrm{NO}}_{3}^{-}$ to NH_4_. A N_2_-fixing cell such as *Halothece* sp. PCC 7418 would rather reduce first the available ${\mathrm{NO}}_{3}^{-}$ than to fix N_2_. Conversely, the N_2_-fixing process is stimulated with low ${\mathrm{NO}}_{3}^{-}$ availability (Manhart and Wong, 1980; Nelson et al., 1982). Since the energy (ATP) to fuel N_2_ fixation is dependent on ${PO}_{4}^{3-}$, the demand for ${PO}_{4}^{3-}$ is theoretically enhanced when the cells are doing N_2_ fixation (in conditions under low ${\mathrm{NO}}_{3}^{-}$ availability). Thus, APase activities are expected to be stimulated under low ${\mathrm{NO}}_{3}^{-}$ conditions, and consequently depend on the availability of Fe because APases such as PhoD may have Fe as co-factor. Moreover, Fe is an important structural component of the nitrogenase enzyme catalyzing the N_2_ fixation process. Nitrogenase contains 38 Fe atoms per holoenzyme since nitrogenase is characterized by slow reaction rates the N_2_-fixers need a large cellular pool of this enzyme, and thus more Fe is needed (Hoffman et al., 2014). The enhanced rates of APase under N_2_-fixing conditions (low ${\mathrm{NO}}_{3}^{-}$ availability) and high Fe availability with low ${PO}_{4}^{3-}$ levels is expected as APases activities are induced with low ${PO}_{4}^{3-}$ levels in the medium (Romano et al., 2017). The control of ${\mathrm{NO}}_{3}^{-}$ and ${PO}_{4}^{3-}$ availabilities in APase activities for N_2_-fixing cells such as *Halothece* sp. PCC 7418 is further supported here with the results of decreased APA rates when ${\mathrm{NO}}_{3}^{-}$, ${PO}_{4}^{3-}$ and Fe were added to cells growing previously with low ${PO}_{4}^{3-}$, low Fe and/or low ${\mathrm{NO}}_{3}^{-}$ levels (Figure 5B).

### Phosphorus and Iron-Uptake and Cellular Contents in *Halothece* sp. PCC 7418

The ${PO}_{4}^{3-}$-uptake measurements in *Halothece* sp. PCC 7418 were done in the experimental units with high ${PO}_{4}^{3-}$ levels because (1) the method used for ${PO}_{4}^{3-}$ analyses was not sensitive enough to measure very low levels of ${PO}_{4}^{3-}$ (≤ 0.1 μM), and (2) APase activities are not induced at high ${PO}_{4}^{3-}$ levels allowing us to evaluate if Fe is also important in ${PO}_{4}^{3-}$ transport mechanisms and not only in APase activities. ${PO}_{4}^{3-}$-uptake rates of *Halothece* sp. PCC 7418 was significantly higher under N_2_-fixing conditions ([Low ${\mathrm{NO}}_{3}^{-}$]) than in non-N_2_ fixing conditions due to the high demand of P for the energy costly N_2_ fixation (Figure 6B). The dependence of N_2_ fixation on P in *Halothece* sp. PCC 7418 is evidenced here with the significant linear correlation between cellular P content of the cells and their rates of N_2_ fixation (Figure 9A), consistent with studies carried out in *Trichodesmium* spp. in the Atlantic (Sañudo-Wilhelmy et al., 2001). In addition, not only N_2_-fixing conditions can enhance the P-requirements of cyanobacteria. It is also reported that under nitrogen limitation, phytoplankton can accumulate carbohydrates and phospholipids, increasing their P-cellular content (Liefer et al., 2019). Different concentrations of Fe, however, did not show significant differences in ${PO}_{4}^{3-}$-uptake at high levels of ${PO}_{4}^{3-}$ availability. This suggests that ${PO}_{4}^{3-}$-uptake mechanisms in this case are not dependent on Fe levels or the Fe present in all treatments (from low to high Fe concentrations) are sufficient for the cells (Figure 6B). The latter case may be most likely since we found significant correlations between the P-cellular and Fe-cellular content of the cells (Figure 9B). These results are also consistent with our data that the highest P-cellular content was found at high Fe levels (Figure 6A), suggesting the narrow connection between P and Fe. The relation between P and Fe cellular contents is also supported by evidences that high concentrations of elemental P are found associated (or co-localized spatially) with Fe within the cells of phytoplankton [*Chlorella* sp. and *Chlamydomonas* sp. (Diaz et al., 2009)]. The Fe-uptake measurements in *Halothece* sp. PCC 7418 in N_2_-fixing conditions revealed that Fe-uptake was correlated with P with high Fe-uptake rates at higher ${PO}_{4}^{3-}$ levels (Figure 8). This may be due to the P-dependence (ATP) of Fe transporters (Noinaj et al., 2010; Kranzler et al., 2013). Results also show the tendency of higher Fe-uptake rates in higher concentrations of Fe in the media, suggesting a passive transport of this metal in *Halothece* sp. PCC 7418. However, this needs to be further investigated.

The time course of depletion of total dissolved phosphate (TDP) in the media (Figure 7A), showed that under ${\mathrm{NO}}_{3}^{-}$ starvation conditions, cells did not deplete TDP, and even increased at the final stage of the experiment suggesting a liberation of cellular TDP of dying cells. Extreme ${\mathrm{NO}}_{3}^{-}$ starvation conditions are suggested here to be detrimental to the growth of *Halothece* sp. PCC 7418 and may have consequences on their P-uptake mechanisms, explaining why APA rates were lower than in [Low ${\mathrm{NO}}_{3}^{-}$] conditions. Even when the nutrients (${PO}_{4}^{3-}$, Fe and/or ${\mathrm{NO}}_{3}^{-}$) were re-inoculated in the cultures that were previously starved with ${\mathrm{NO}}_{3}^{-}$, the cells did not acclimate and were not capable of depleting TDP from the media (Figure 7B). Whereas, much of the previous research has focused on the inhibition or sensitivity of N_2_ fixation to increased availability of dissolved inorganic nitrogen (e.g., ${\mathrm{NO}}_{3}^{-}$, NH_4_^+^) (Knapp, 2012), investigations on the physiological conditions for growth of N_2_-fixers are few. Spiller and Shanmugam (1987), gave some evidences that a unicellular species of marine N_2_-fixer *Synechococcus* sp. strain SF1 (isolated from macroalgae, *Sargassum fluitans*) is dependent on the presence and type of carbon (C) source to support its growth with N_2_ as the sole nitrogen source. Their results showed, for example, that without the addition of C source (e.g., HCO_3_^−^), there was no growth of the species tested with N_2_ as the sole source. Moreover, some studies have reported less cell yield of unicellular N_2_ fixers when grown with N_2_ as sole N source compared with addition of ${\mathrm{NO}}_{3}^{-}$ since N_2_ fixation is an energetically costly process (Spiller and Shanmugam, 1987; Agawin et al., 2007). Our result that extreme ${\mathrm{NO}}_{3}^{-}$ starvation condition (at nM levels close to N_2_ as sole source) is suggested to be detrimental to the growth of *Halothece* sp. PCC 7418 may be due to the type of C source (glucose and citrate) in our treatments which may not be the optimum for growth of this species with N_2_ as sole N source. This hypothesis however needs more investigations.

In summary, this is the first study investigating the interaction between ${PO}_{4}^{3-}$, Fe, and ${\mathrm{NO}}_{3}^{-}$ availabilities in the P-acquisition mechanisms of a unicellular N_2_-fixing bacteria found in association with the Mediterranean seagrass *P*. *oceanica*. The results suggest that APase activities under inorganic P-limited conditions are enhanced by increased Fe availabilities. The ${PO}_{4}^{3-}$ and Fe dependence of *Halothece* sp. PCC 7418 depends whether they are grown in N_2_-fixing conditions (i.e., low ${\mathrm{NO}}_{3}^{-}$ levels) or not. Genomic and structural analyses have also shown the tight association between P-acquisition mechanisms and Fe in *Halothece* sp. PCC 7418. Studies combining genomic and protein structural analyses and experimental approaches are important to investigate in detail the control of environmental factors (e.g., availability of metals and nutrients) to the functioning of N_2_-fixing organisms found in important species of seagrasses.

## Data Availability

The raw data supporting the conclusions of this manuscript will be made available by the authors, without undue reservation, to any qualified researcher.

## Author Contributions

VF-J and NA designed the experiments. VF-J conducted all experiments and led the writing of the paper. All authors contributed to the writing and review of the manuscript, and NA is the supervisor of the laboratory.

### Conflict of Interest Statement

The authors declare that the research was conducted in the absence of any commercial or financial relationships that could be construed as a potential conflict of interest.
