# Supplementary material for: The Role of Iron in the P-Acquisition Mechanisms of the Unicellular N2-Fixing Cyanobacteria Halothece sp., Found in Association With the Mediterranean Seagrass Posidonia oceanica
Source: Front Microbiol. 2019 Aug 22;10:1903. doi: 10.3389/fmicb.2019.01903 (PMC6713934; doi:10.3389/fmicb.2019.01903)
Supplement: Supplementary file 1 [file Table_1.DOCX]

**Supplementary Table 1**. All genes and Locus Tags of the predicted Pho regulon of all the tested strains.

| ***Nostoc punctiforme* PCC 73102 (CP001037)** | | | ***Chroococcidiopsis thermalis* PCC 7203 (CP003598)** | | |
| --- | --- | --- | --- | --- | --- |
| **Genes** | **Annotation (Patric)** | **Locus Tag** | **Genes** | **Annotation (Patric)** | **Locus Tag** |
| *phoU* | - | - | *phoU* | - | - |
| *pstS* | - | - | *pstS* | - | - |
| *pstC* | - | - | *pstC* | - | - |
| *pstA* | - | - | *pstA* | - | - |
| *pstB* | - | - | *pstB* | - | - |
| *phoR* | - | - | *phoR* | Phosphate regulon sensor protein PhoR (SphS) | Chro_5919 |
| *phoB* | - | - | *phoB* | - | - |
| APase | - | - | APase | Alkaline phosphatase | Chro_5919 |
| *ppK* | - | - | *ppK* | - | - |
| *ppX* | - | - | *ppX* | - | - |
| *ppA* | - | - | *ppA* | - | - |
| ***Spirulina subsalsa* PCC 9445 (NZ_ALVR01000000)** | | | ***Crocosphaera watsonii* WH 8501** (**NZ_AADV02000000)** | | |
| **Genes** | **Annotation (Patric)** | **Locus Tag** | **Genes** | **Annotation (Patric)** | **Locus Tag** |
| *phoU* | Phosphate transport system regulatory protein PhoU | SPI9445_RS0119485 | *phoU* | Phosphate transport system regulatory protein PhoU | CwatDRAFT_5911 |
| *pstS* | Phosphate ABC transporter, substrate-binding protein PstS | SPI9445_RS0105090 | *pstS* | Phosphate ABC transporter, substrate-binding protein PstS | CwatDRAFT_6534 |
|  | Phosphate ABC transporter, substrate-binding protein PstS | SPI9445_RS0120490 |  | Phosphate ABC transporter, substrate-binding protein PstS | CwatDRAFT_5160 |
|  | Phosphate ABC transporter, substrate-binding protein PstS | SPI9445_RS0121920 |  | Phosphate ABC transporter, substrate-binding protein PstS | CwatDRAFT_4928 |
| *pstC* | Phosphate ABC transporter, permease protein PstC | SPI9445_RS0120485 | *pstC* | Phosphate ABC transporter, permease protein PstC | CwatDRAFT_4929 |
| *pstA* | Phosphate ABC transporter, permease protein PstA | SPI9445_RS0120480 | *pstA* | Phosphate ABC transporter, permease protein PstA | CwatDRAFT_4930 |
| *pstB* | Phosphate ABC transporter, permease protein PstB | SPI9445_RS0107060 |  | Phosphate ABC transporter, permease protein PstA | CwatDRAFT_4931 |
| *phoR* | Phosphate regulon sensor protein PhoR (SphS) | SPI9445_RS0119490 | *pstB* | Phosphate ABC transporter, permease protein PstB | CwatDRAFT_4932 |
| *phoB* | Phosphate regulon transcriptional regulatory protein PhoB (SphR) | SPI9445_RS0119495 |  | Phosphate ABC transporter, permease protein PstB | CwatDRAFT_4933 |
| APase | - | - |  | Phosphate ABC transporter, permease protein PstB | CwatDRAFT_1348 |
| *ppK* | Polyphosphate kinase | SPI9445_RS0118550 | *phoR* | Phosphate regulon sensor protein PhoR (SphS) | CwatDRAFT_5910 |
| *ppX* | Exopolyphosphatase | SPI9445_RS0101940 | *phoB* | Phosphate regulon transcriptional regulatory protein PhoB (SphR) | CwatDRAFT_2775 |
| *ppA* | Inorganic pyrophosphatase | SPI9445_RS0119415 | APase | Alkaline phosphatase | CwatDRAFT_1549 |
|  | | |  | Alkaline phosphatase | CwatDRAFT_1629 |
|  |  |  | *ppK* | Polyphosphate kinase | CwatDRAFT_6491 |
|  |  |  | *ppX* | Exopolyphosphatase | CwatDRAFT_1948 |
|  |  |  | *ppA* | Inorganic pyrophosphatase | CwatDRAFT_2235 |
| ***Synechocystis* sp. PCC 7509 (NZ_ALVU02000000)** | | | ***Pleurocapsa* sp. PCC 7327 (CP003590)** | | |
| **Genes** | **Annotation (Patric)** | **Locus Tag** | **Genes** | **Annotation (Patric)** | **Locus Tag** |
| *phoU* | Phosphate transport system regulatory protein PhoU | SYN7509_RS0215960 | *phoU* | Phosphate transport system regulatory protein PhoU | Ple7327_4124 |
| *pstS* | Phosphate ABC transporter, substrate-binding protein PstS | SYN7509_RS0206650 | *pstS* | Phosphate ABC transporter, substrate-binding protein PstS | Ple7327_1250 |
|  | Phosphate ABC transporter, substrate-binding protein PstS | SYN7509_RS0207360 |  | Phosphate ABC transporter, substrate-binding protein PstS | Ple7327_3388 |
|  | Phosphate ABC transporter, substrate-binding protein PstS | SYN7509_RS0215920 |  | Phosphate ABC transporter, substrate-binding protein PstS | Ple7327_3389 |
|  | Phosphate ABC transporter, substrate-binding protein PstS | SYN7509_RS0215925 |  | Phosphate ABC transporter, substrate-binding protein PstS | Ple7327_3618 |
| *pstC* | Phosphate ABC transporter, permease protein PstC | SYN7509_RS0206655 | *pstC* | Phosphate ABC transporter, permease protein PstC | Ple7327_1249 |
|  | Phosphate ABC transporter, permease protein PstC | SYN7509_RS0207365 |  | Phosphate ABC transporter, permease protein PstC | Ple7327_3387 |
| *pstA* | Phosphate ABC transporter, permease protein PstA | SYN7509_RS0206660 | *pstA* | Phosphate ABC transporter, permease protein PstA | Ple7327_1248 |
|  | Phosphate ABC transporter, permease protein PstA | SYN7509_RS0207370 |  | Phosphate ABC transporter, permease protein PstA | Ple7327_3386 |
| *pstB* | Phosphate ABC transporter, permease protein PstB | SYN7509_RS0206665 | *pstB* | Phosphate ABC transporter, permease protein PstB | Ple7327_1247 |
|  | Phosphate ABC transporter, permease protein PstB | SYN7509_RS0207375 |  | Phosphate ABC transporter, permease protein PstB | Ple7327_3385 |
| *phoR* | Phosphate regulon sensor protein PhoR (SphS) | SYN7509_RS0215965 | *phoR* | Phosphate regulon sensor protein PhoR (SphS) | Ple7327_4123 |
| *phoB* | Phosphate regulon transcriptional regulatory protein PhoB (SphR) | SYN7509_RS0215970 | *phoB* | Phosphate regulon transcriptional regulatory protein PhoB (SphR) | Ple7327_4122 |
| APase | Alkaline phosphatase | SYN7509_RS0210500 | APase | Phosphodiesterase/alkaline phosphatase D | Ple7327_0042 |
| *ppK* | Polyphosphate kinase | SYN7509_RS0202140 |  | Alkaline phosphatase | Ple7327_1998 |
|  | Polyphosphate kinase 2 | SYN7509_RS0205545 |  | Alkaline phosphatase like protein | Ple7327_4613 |
| *ppX* | Exopolyphosphatase | SYN7509_RS0215695 | *ppK* | Polyphosphate kinase | Ple7327_2011 |
| *ppA* | Inorganic pyrophosphatase | SYN7509_RS0218560 | *ppX* | Exopolyphosphatase | Ple7327_1164 |
|  | | | *ppA* | Inorganic pyrophosphatase | Ple7327_3589 |
| ***Anabaena* sp. PCC 7108 (NZ_AJWF01000000)** | | | ***Dactylococcopsis salina* PCC 8305 (CP003944)** | | |
| **Genes** | **Annotation (Patric)** | **Locus Tag** | **Genes** | **Annotation (Patric)** | **Locus Tag** |
| *phoU* | Phosphate transport system regulatory protein PhoU | ANA7108_RS010733 | *phoU* | Phosphate transport system regulatory protein PhoU | Dacsa_1982 |
| *pstS* | Phosphate ABC transporter, substrate-binding protein PstS | ANA7108_RS0104250 | *pstS* | Phosphate ABC transporter, substrate-binding protein PstS | Dacsa_0981 |
|  | Phosphate ABC transporter, substrate-binding protein PstS | ANA7108_RS0114535 |  | Phosphate ABC transporter, substrate-binding protein PstS | Dacsa_1219 |
|  | Phosphate ABC transporter, substrate-binding protein PstS | ANA7108_RS0124135 |  | Phosphate ABC transporter, substrate-binding protein PstS | Dacsa_1417 |
|  | Phosphate ABC transporter, substrate-binding protein PstS | ANA7108_RS0125995 |  | Phosphate ABC transporter, substrate-binding protein PstS | Dacsa_1575 |
| *pstC* | Phosphate ABC transporter, permease protein PstC | ANA7108_RS0114530 |  | Phosphate ABC transporter, substrate-binding protein PstS | Dacsa_3076 |
|  | Phosphate ABC transporter, permease protein PstC | ANA7108_RS0124130 | *pstC* | Phosphate ABC transporter, permease protein PstC | Dacsa_1220 |
| *pstA* | Phosphate ABC transporter, permease protein PstA | ANA7108_RS0114525 |  | Phosphate ABC transporter, permease protein PstC | Dacsa_1416 |
|  | Phosphate ABC transporter, permease protein PstA | ANA7108_RS0124125 |  | Phosphate ABC transporter, permease protein PstC | Dacsa_3114 |
| *pstB* | Phosphate ABC transporter, permease protein PstB | ANA7108_RS0114520 | *pstA* | Phosphate ABC transporter, permease protein PstA | Dacsa_1224 |
|  | Phosphate ABC transporter, permease protein PstB | ANA7108_RS0111425 |  | Phosphate ABC transporter, permease protein PstA | Dacsa_1415 |
|  | Phosphate ABC transporter, permease protein PstB | ANA7108_RS0124120 | *pstB* | Phosphate ABC transporter, permease protein PstB | Dacsa_1414 |
| *phoR* | Phosphate regulon sensor protein PhoR (SphS) | ANA7108_RS0107330 |  | Phosphate ABC transporter, permease protein PstB | Dacsa_3115 |
| *phoB* | Phosphate regulon transcriptional regulatory protein PhoB (SphR) | ANA7108_RS0107325 | *phoR* | Phosphate regulon sensor protein PhoR (SphS) | Dacsa_1981 |
| Apase | Alkaline phosphatase | ANA7108_RS0104615 | *phoB* | Phosphate regulon transcriptional regulatory protein PhoB (SphR) | Dacsa_1980 |
|  | Alkaline phosphatase | ANA7108_RS0104600 | APase | Alkaline phosphatase | Dacsa_0439 |
|  | Alkaline phosphatase | ANA7108_RS0100780 |  | Alkaline phosphatase | Dacsa_1692 |
|  | Alkaline phosphatase | ANA7108_RS0111735 |  | Alkaline phosphatase | Dacsa_1693 |
| *ppK* | Polyphosphate kinase 2 | ANA7108_RS0119175 |  | Alkaline phosphatase | Dacsa_1695 |
|  | Polyphosphate kinase | ANA7108_RS0109985 |  | Alkaline phosphatase | Dacsa_1696 |
| *ppX* | Exopolyphosphatase | ANA7108_RS0118330 |  | Alkaline phosphatase | Dacsa_2870 |
| *ppA* | Inorganic pyrophosphatase | ANA7108_RS0101510 | *ppK* | Polyphosphate kinase | Dacsa_1689 |
|  | | | *ppX* | Exopolyphosphatase | Dacsa_0957 |
|  |  |  | *ppA* | Inorganic pyrophosphatase | Dacsa_3296 |
| ***Microcystis aeruginosa* PCC 7806SL (CP020771)** | | | ***Fischerella* sp. PCC 9339 (NZ_ALVS01000000)** | | |
| **Genes** | **Annotation (Patric)** | **Locus Tag** | **Genes** | **Annotation (Patric)** | **Locus Tag** |
| *phoU* | Phosphate transport system regulatory protein PhoU | BH695_5315 | *phoU* | Phosphate transport system regulatory protein PhoU | PCC9339_RS0124490 |
| *pstS* | Phosphate ABC transporter, substrate-binding protein PstS | BH695_0746 | *pstS* | Phosphate ABC transporter, substrate-binding protein PstS | PCC9339_RS0107315 |
|  | Phosphate ABC transporter, substrate-binding protein PstS | BH695_0747 |  | Phosphate ABC transporter, substrate-binding protein PstS | PCC9339_RS0115315 |
|  | Phosphate ABC transporter, substrate-binding protein PstS | BH695_1907 |  | Phosphate ABC transporter, substrate-binding protein PstS | PCC9339_RS0118145 |
|  | Phosphate ABC transporter, substrate-binding protein PstS | BH695_1911 |  | Phosphate ABC transporter, substrate-binding protein PstS | PCC9339_RS0124050 |
|  | Phosphate ABC transporter, substrate-binding protein PstS | BH695_4434 |  | Phosphate ABC transporter, substrate-binding protein PstS | PCC9339_RS013259 |
| *pstC* | Phosphate ABC transporter, permease protein PstC | BH695_0748 | *pstC* | Phosphate ABC transporter, permease protein PstC | PCC9339_RS0107310 |
|  | Phosphate ABC transporter, permease protein PstC | BH695_1910 |  | Phosphate ABC transporter, permease protein PstC | PCC9339_RS0124055 |
| *pstA* | Phosphate ABC transporter, permease protein PstA | BH695_0749 |  | Phosphate ABC transporter, permease protein PstC | PCC9339_RS0132595 |
|  | Phosphate ABC transporter, permease protein PstA | BH695_1909 | *pstA* | Phosphate ABC transporter, permease protein PstA | PCC9339_RS0107305 |
| *pstB* | Phosphate ABC transporter, permease protein PstB | BH695_0750 |  | Phosphate ABC transporter, permease protein PstA | PCC9339_RS0124060 |
|  | Phosphate ABC transporter, permease protein PstB | BH695_0752 |  | Phosphate ABC transporter, permease protein PstA | PCC9339_RS0132600 |
|  | Phosphate ABC transporter, permease protein PstB | BH695_1908 | *pstB* | Phosphate ABC transporter, permease protein PstB | PCC9339_RS0107300 |
| *phoR* | Phosphate regulon sensor protein PhoR (SphS) | BH695_3368 |  | Phosphate ABC transporter, permease protein PstB | PCC9339_RS0124065 |
|  | Phosphate regulon sensor protein PhoR (SphS) | BH695_5316 |  | Phosphate ABC transporter, permease protein PstB | PCC9339_RS0132605 |
| *phoB* | Phosphate regulon transcriptional regulatory protein PhoB (SphR) | BH695_5317 | *phoR* | Phosphate ABC transporter, permease protein PstB | PCC9339_RS0124065 |
| Apase | Alkaline phosphatase | BH695_2564 | *phoB* | Phosphate ABC transporter, permease protein PstB | PCC9339_RS0132605 |
|  | Alkaline phosphatase | BH695_2565 | Apase | Alkaline phosphatase | PCC9339_RS0114510 |
|  | Alkaline phosphatase | BH695_2649 |  | Alkaline phosphatase | PCC9339_RS0133125 |
|  | Alkaline phosphatase | BH695_3440 |  | Alkaline phosphatase | PCC9339_RS0120820 |
|  | Alkaline phosphatase-like protein | BH695_4984 | *ppK* | Polyphosphate kinase | PCC9339_RS0116345 |
| *ppK* | Polyphosphate kinase | BH695_2980 |  | Polyphosphate kinase 2 | PCC9339_RS0129015 |
| *ppX* | Exopolyphosphatase | BH695_0037 |  | Polyphosphate kinase | PCC9339_RS0129835 |
|  | Exopolyphosphatase | BH695_5350 | *ppX* | Exopolyphosphatase | PCC9339_RS0123895 |
| *ppA* | Inorganic pyrophosphatase | BH695_0664 | *ppA* | Inorganic pyrophosphatase | PCC9339_RS0115895 |
| ***Halothece* sp. PCC 7418 (CP003945)** | | | ***Cyanothece* sp. ATCC 51472 (NZ_AGJC02000000)** | | |
| **Genes** | **Annotation (Patric)** | **Locus Tag** | **Genes** | **Annotation (Patric)** | **Locus Tag** |
| *phoU* | Phosphate transport system regulatory protein PhoU | PCC7418_3123 | *phoU* | Phosphate transport system regulatory protein PhoU | CY51472DRAFT_RS0222590 |
| *pstS* | Phosphate ABC transporter, substrate-binding protein PstS | PCC7418_0041 | *pstS* | Phosphate ABC transporter, substrate-binding protein PstS | CY51472DRAFT_RS0201580 |
|  | Phosphate ABC transporter, substrate-binding protein PstS | PCC7418_0390 |  | Phosphate ABC transporter, substrate-binding protein PstS | CY51472DRAFT_RS0209720 |
|  | Phosphate ABC transporter, substrate-binding protein PstS | PCC7418_1750 |  | Phosphate ABC transporter, substrate-binding protein PstS | CY51472DRAFT_RS0209725 |
|  | Phosphate ABC transporter, substrate-binding protein PstS | PCC7418_3269 |  | Phosphate ABC transporter, substrate-binding protein PstS | CY51472DRAFT_RS0210980 |
| *pstC* | Phosphate ABC transporter, permease protein PstC | PCC7418_1749 |  | Phosphate ABC transporter, substrate-binding protein PstS | CY51472DRAFT_RS0211095 |
|  | Phosphate ABC transporter, permease protein PstC | PCC7418_1912 |  | Phosphate ABC transporter, substrate-binding protein PstS | CY51472DRAFT_RS0214500 |
|  | Phosphate ABC transporter, permease protein PstC | PCC7418_3270 |  | Phosphate ABC transporter, substrate-binding protein PstS | CY51472DRAFT_RS0224580 |
| *pstA* | Phosphate ABC transporter, permease protein PstA | PCC7418_1748 | *pstC* | Phosphate ABC transporter, permease protein PstC | CY51472DRAFT_RS0209715 |
|  | Phosphate ABC transporter, permease protein PstA | PCC7418_3271 |  | Phosphate ABC transporter, permease protein PstC | CY51472DRAFT_RS0211100 |
| *pstB* | Phosphate ABC transporter, permease protein PstB | PCC7418_1747 | *pstA* | Phosphate ABC transporter, permease protein PstA | CY51472DRAFT_RS0209710 |
|  | Phosphate ABC transporter, permease protein PstB | PCC7418_1911 |  | Phosphate ABC transporter, permease protein PstA | CY51472DRAFT_RS0211105 |
|  | Phosphate ABC transporter, permease protein PstB | PCC7418_3272 | *pstB* | Phosphate ABC transporter, permease protein PstB | CY51472DRAFT_RS0201590 |
| *phoR* | Phosphate regulon sensor protein PhoR (SphS) | PCC7418_3124 |  | Phosphate ABC transporter, permease protein PstB | CY51472DRAFT_RS0224545 |
| *phoB* | Phosphate regulon transcriptional regulatory protein PhoB (SphR) | PCC7418_3124 |  | Phosphate ABC transporter, permease protein PstB | CY51472DRAFT_RS0209700 |
| Apase | Alkaline phosphatase | PCC7418_0071 |  | Phosphate ABC transporter, permease protein PstB | CY51472DRAFT_RS0211110 |
|  | Alkaline phosphatase | PCC7418_0077 |  | Phosphate ABC transporter, permease protein PstB | CY51472DRAFT_RS0209705 |
|  | Alkaline phosphatase | PCC7418_0571 |  | Phosphate ABC transporter, permease protein PstB | CY51472DRAFT_RS0224540 |
|  | Alkaline phosphatase | PCC7418_1065 | *phoR* | Phosphate regulon sensor protein PhoR (SphS) | CY51472DRAFT_RS0221650 |
|  | Phosphodiesterase/alkaline phosphatase D | PCC7418_1982 | *phoB* | Phosphate regulon transcriptional regulatory protein PhoB (SphR) | CY51472DRAFT_RS0201450 |
|  | Alkaline phosphatase | PCC7418_2858 | Apase | Alkaline phosphatase | CY51472DRAFT_RS0203850 |
|  | Alkaline phosphatase | PCC7418_3483 |  | Alkaline phosphatase | CY51472DRAFT_RS0203450 |
|  | Alkaline phosphatase | PCC7418_3485 |  | Alkaline phosphatase | CY51472DRAFT_RS0222455 |
| *ppK* | Polyphosphate kinase | PCC7418_1073 |  | Alkaline phosphatase | CY51472DRAFT_RS0204765 |
| *ppX* | Exopolyphosphatase | PCC7418_2036 | *ppK* | Polyphosphate kinase | CY51472DRAFT_RS0216060 |
| *ppA* | Inorganic pyrophosphatase | PCC7418_0708 |  | Polyphosphate kinase | CY51472DRAFT_RS0224465 |
|  | | | *ppX* | Exopolyphosphatase | CY51472DRAFT_RS0208320 |
|  |  |  | *ppA* | Inorganic pyrophosphatase | CY51472DRAFT_RS0203230 |
| ***Calothrix* sp. PCC 7507 (CP003943)** | | | ***Gloeocapsa* sp. PCC 7428 (CP003646)** | | |
| **Genes** | **Annotation (Patric)** | **Locus Tag** | **Genes** | **Annotation (Patric)** | **Locus Tag** |
| *phoU* | Phosphate transport system regulatory protein PhoU | Cal7507_1625 | *phoU* | Phosphate transport system regulatory protein PhoU | Glo7428_3037 |
| *pstS* | Phosphate ABC transporter, substrate-binding protein PstS | Cal7507_0340 | *pstS* | Phosphate ABC transporter, substrate-binding protein PstS | Glo7428_0758 |
|  | Phosphate ABC transporter, substrate-binding protein PstS | Cal7507_1013 |  | Phosphate ABC transporter, substrate-binding protein PstS | Glo7428_0759 |
|  | Phosphate ABC transporter, substrate-binding protein PstS | Cal7507_1206 |  | Phosphate ABC transporter, substrate-binding protein PstS | Glo7428_1132 |
|  | Phosphate ABC transporter, substrate-binding protein PstS | Cal7507_1927 |  | Phosphate ABC transporter, substrate-binding protein PstS | Glo7428_1752 |
|  | Phosphate ABC transporter, substrate-binding protein PstS | Cal7507_1928 |  | Phosphate ABC transporter, substrate-binding protein PstS | Glo7428_1886 |
|  | Phosphate ABC transporter, substrate-binding protein PstS | Cal7507_4798 |  | Phosphate ABC transporter, substrate-binding protein PstS | Glo7428_2342 |
|  | Phosphate ABC transporter, substrate-binding protein PstS | Cal7507_5691 |  | Phosphate ABC transporter, substrate-binding protein PstS | Glo7428_4983 |
|  | Phosphate ABC transporter, substrate-binding protein PstS | Cal7507_5816 | *pstC* | Phosphate ABC transporter, permease protein PstC | Glo7428_1133 |
| *pstC* | Phosphate ABC transporter, permease protein PstC | Cal7507_0341 |  | Phosphate ABC transporter, permease protein PstC | Glo7428_2341 |
|  | Phosphate ABC transporter, permease protein PstC | Cal7507_1207 |  | Phosphate ABC transporter, permease protein PstC | Glo7428_4984 |
|  | Phosphate ABC transporter, permease protein PstC | Cal7507_4797 | *pstA* | Phosphate ABC transporter, permease protein PstA | Glo7428_1134 |
| *pstA* | Phosphate ABC transporter, permease protein PstA | Cal7507_0342 |  | Phosphate ABC transporter, permease protein PstA | Glo7428_2340 |
|  | Phosphate ABC transporter, permease protein PstA | Cal7507_1208 |  | Uncharacterized protein Psta_3961 | Glo7428_3942 |
|  | Phosphate ABC transporter, permease protein PstA | Cal7507_4796 |  | Phosphate ABC transporter, permease protein PstA | Glo7428_4985 |
| *pstB* | Phosphate ABC transporter, permease protein PstB | Cal7507_0343 | *pstB* | Phosphate ABC transporter, permease protein PstB | Glo7428_1135 |
|  | Phosphate ABC transporter, permease protein PstB | Cal7507_1209 |  | Phosphate ABC transporter, permease protein PstB | Glo7428_2339 |
|  | Phosphate ABC transporter, permease protein PstB | Cal7507_1210 | *phoR* | Phosphate regulon sensor protein PhoR (SphS) | Glo7428_0220 |
|  | Phosphate ABC transporter, permease protein PstB | Cal7507_4795 |  | Phosphate regulon sensor protein PhoR (SphS) | Glo7428_3038 |
| *phoR* | Phosphate regulon sensor protein PhoR (SphS) | Cal7507_1624 |  | Phosphate regulon sensor protein PhoR (SphS) | Glo7428_5081 |
| *phoB* | Phosphate regulon transcriptional regulatory protein PhoB (SphR) | Cal7507_1623 | *phoB* | Phosphate regulon transcriptional regulatory protein PhoB (SphR) | Glo7428_0934 |
| Apase | Alkaline phosphatase | Cal7507_1562 |  | Phosphate regulon transcriptional regulatory protein PhoB (SphR) | Glo7428_3039 |
|  | Alkaline phosphatase | Cal7507_2517 | Apase | Alkaline phosphatase | Glo7428_0109 |
|  | Alkaline phosphatase | Cal7507_2523 |  | Alkaline phosphatase | Glo7428_0424 |
| *ppK* | Polyphosphate kinase | Cal7507_0098 |  | Alkaline phosphatase | Glo7428_0645 |
|  | Polyphosphate kinase | Cal7507_2880 |  | Alkaline phosphatase | Glo7428_0796 |
|  | Polyphosphate kinase | Cal7507_4641 |  | Alkaline phosphatase | Glo7428_0846 |
| *ppX* | Exopolyphosphatase | Cal7507_4902 |  | Alkaline phosphatase | Glo7428_1248 |
| *ppA* | Inorganic pyrophosphatase | Cal7507_2098 |  | Alkaline phosphatase | Glo7428_1514 |
|  | | |  | Alkaline phosphatase | Glo7428_1646 |
|  |  |  |  | Alkaline phosphatase | Glo7428_1647 |
|  |  |  |  | Phosphodiesterase/alkaline phosphatase D | Glo7428_3016 |
|  |  |  |  | Alkaline phosphatase | Glo7428_3293 |
|  |  |  |  | Alkaline phosphatase | Glo7428_3370 |
|  |  |  |  | Alkaline phosphatase | Glo7428_3554 |
|  |  |  |  | Alkaline phosphatase | Glo7428_3943 |
|  |  |  |  | Alkaline phosphatase | Glo7428_4190 |
|  |  |  |  | Alkaline phosphatase | Glo7428_4312 |
|  |  |  |  | Alkaline phosphatase | Glo7428_4364 |
|  |  |  |  | Alkaline phosphatase | Glo7428_4414 |
|  |  |  |  | Phosphodiesterase/alkaline phosphatase D | Glo7428_4942 |
|  |  |  | *ppK* | Polyphosphate kinase | Glo7428_1568 |
|  |  |  |  | Polyphosphate kinase 2 | Glo7428_1966 |
|  |  |  | *ppX* | Exopolyphosphatase | Glo7428_1713 |
|  |  |  | *ppA* | Inorganic pyrophosphatase | Glo7428_1953 |
